# Reconstructing Molecular Networks by Causal Diffusion Do‐Calculus Analysis with Deep Learning

**DOI:** 10.1002/advs.202409170

**Published:** 2024-10-23

**Authors:** Jiachen Wang, Yuelei Zhang, Luonan Chen, Xiaoping Liu

**Affiliations:** ^1^ Key Laboratory of Systems Health Science of Zhejiang Province School of Life Science Hangzhou Institute for Advanced Study University of Chinese Academy of Sciences Hangzhou 310024 China; ^2^ Key Laboratory of Systems Biology Shanghai Institute of Biochemistry and Cell Biology Center for Excellence in Molecular Cell Science Chinese Academy of Sciences Shanghai 200031 China

**Keywords:** causal network inference, diffusion model, do‐calculus, gene regulatory networks

## Abstract

Quantifying molecular regulations between genes/molecules causally from observed data is crucial for elucidating the molecular mechanisms underlying biological processes at the network level. Presently, most methods for inferring gene regulatory and biological networks rely on association studies or observational causal‐analysis approaches. This study introduces a novel approach that combines intervention operations and diffusion models within a do‐calculus framework by deep learning, i.e., Causal Diffusion Do‐calculus (CDD) analysis, to infer causal networks between molecules. CDD can extract causal relations from observed data owing to its intervention operations, thereby significantly enhancing the accuracy and generalizability of causal network inference. Computationally, CDD has been applied to both simulated data and real omics data, which demonstrates that CDD outperforms existing methods in accurately inferring gene regulatory networks and identifying causal links from genes to disease phenotypes. Especially, compared with the Mendelian randomization algorithm and other existing methods, the CDD can reliably identify the disease genes or molecules for complex diseases with better performances. In addition, the causal analysis between various diseases and the potential factors in different populations from the UK Biobank database is also conducted, which further validated the effectiveness of CDD.

## Graphical abstract

Causal Diffusion Do‐calculus (CDD) is a new method that combines diffusion models with do‐calculus to infer causality between observed variables. Compared with Granger causality (GC) and Mendelian randomization (MR), CDD has a broader range of applications and can infer causal relationships only based on observed data. Furthermore, unlike traditional biological network inference methods such as GENIE3, CDD is grounded in causal theory for its causal inference.

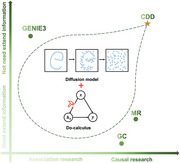

## 1 Introduction

Understanding cause‐and‐effect relationships is crucial to elucidate the molecular mechanisms of biological processes at a network level. The core of network inference such as gene regulatory networks or protein interaction networks, and disease‐driven factor inference from genes/molecules to disease phenotypes, fundamentally revolves around causality.^[^ ^1^, ^2^ ^]^ Traditional methods in network inference, including tools like GENIE3, PLSNet, and others^[^ ^3^, ^4^, ^5^ ^]^ for gene regulatory networks, often rely on statistical methods or association studies without considering causality, thus often leading to inaccuracies and spurious correlations. Other methods, such as CellOracle,^[^ ^6^ ^]^ require specific a priori knowledge, like identifying transcription factors (TF) and their targets. The scMTNI requires other information, such as an assay for transposase‐accessible chromatin using sequencing (ATAC‐seq).^[^ ^7^ ^]^ While effective in their domains, these approaches lack universality and struggle without prior knowledge, limiting their applicability to network reconstruction from the observed data. On the other hand, for causal inference from genotypes/molecules to phenotypes,^[^ ^8^ ^]^ the most widely‐used method is Mendelian randomization,^[^ ^2^, ^9^, ^10^ ^]^ which bridges the genotypes‐phenotype problem through SNP loci. However, this method is primarily constrained to SNP analysis and struggles with other multivariate problems such as the high risk of false negative results. In the traditional field of causal inference, Granger causality^[^ ^11^, ^12^ ^]^ is indeed a commonly used method. However, Granger causality is primarily applicable to time series data and may not be well‐suited for non‐time series data commonly encountered in biology. Granger causality is limited to time series data, making it unsuitable for many biological datasets. Another frequently used method is the additive noise model (ANM)^[^ ^13^ ^]^ which is often employed to assess causal identifiability. The ANM focuses on determining causal relationships between a pair of variables by incorporating additive noise terms. However, the ANM typically considers only a single pair of variables and may not fully account for the influence of other variables in the system. CVP is a way to infer genetic cause and effect,^[^ ^14^ ^]^ but it can only determine linear relationships. Recent rapid advances in causal inference^[^ ^15^, ^16^ ^]^ such as intervention (do calculus) operations open a new way for causality analysis. Here, we proposed a novel approach, Causal Diffusion Do‐calculus (CDD), combining diffusion models and do‐calculus for accurate causal inference. To alleviate the complexities or nonlinearity inherent in gene regulation, the diffusion model demonstrates strong capabilities in modeling the underlying causal regulatory relationships. Many methods rely heavily on TF sites to provide causality, so these methods cannot infer causality without exploiting Supporting Information. Our approach, rooted firmly in interventional causation rather than mere association or observational causation, significantly enhances the accuracy and generalizability of network inference even without TF information. Furthermore, by performing intervention operations on the hidden layer of the diffusion model, we can achieve the manipulation of the input genes by the do‐calculus. Since the forward process of the diffusion model can be realized end‐to‐end, such intervention operations on the hidden layer theoretically can effectively reflect the causal manipulation of the input genes. Computationally, our CDD method has been rigorously tested on both simulated and real omics data. These applications have demonstrated CDD's superiority in not only inferring gene regulatory networks but also identifying causal links from genes to disease phenotypes, outperforming the Mendelian randomization algorithm and other existing methods. In particular, we also conducted the causal analysis between various diseases and the potential factors in different populations from the UK Biobank database, which further validated the effectiveness of CDD. Both theoretical and computational results demonstrate that our approach marks a significant advancement in the field of biological network analysis, providing a more reliable, causation‐focused lens for deciphering the complexities of molecular interactions.

## 2 Results

### 2.1 Main Theoretical Result of Interventional Causality in CDD

**Figure 1:**
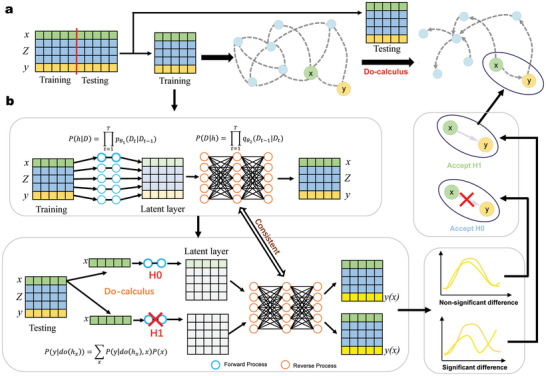
Schematic representation of Causal Diffusion Do‐calculus (CDD) model. a) The flowchart of the CDD algorithm includes H0 and H1 assumptions, diffusion model and do‐calculus (CDD model), and causality decision. b) The diffusion model generates the forward and backward process functions. Then, do‐calculus generates *y* under the intervention state through the intervention operation of the hidden layer. Finally, according to the assumptions of H0 and H1, depending on whether or not there is a significant difference, we can determine the causality. H0 and H1 are respectively whether there is a causal relationship from *x* to *y*.

In **Figure** **1**b, as stated in Materials and Methods, we assume variables *D* = (*x*, *Z*, *y*)  ∈ *R^n^* as the data domain. Their corresponding hidden layer representations are given by *H*  =  (*h_x_*, *h_Z_*, *h_y_*) ∈ *R^n^*. Then, we have the following proposition.

Proposition 1. For any *D* ⊥ ξ where ξ represents Gaussian noise and ⊥ means the independence in Figure 1, the set of variables *x* satisfies the backdoor criterion with respect to (*h_x_*, *y*).

The detailed proof of this proposition is provided in Section S1 (Supporting Information). Then, with do‐calculus (7) on the hidden layer variable *h_x_* in our CDD algorithm (Figure 1), we can determine the causal relation from *x* to *y* in the sense of Judea Pearl causality.^[^ ^17^ ^]^ Next, our algorithm was applied to first infer gene regulatory networks and then disease‐phenotype relationships in SNP data. Our results demonstrate the effectiveness of CDD in identifying causal relationships across various types of data.

### 2.2 Causal Inference of Gene Regulatory Networks

We first tackled the gene regulatory network inference problem and conducted a comparative analysis with other algorithms. Specifically, we selected GENIE3^[^ ^4^ ^]^ and PLSNet,^[^ ^5^ ^]^ ODEs^[^ ^18^ ^]^ as the methods for comparison, with GENIE3 being the most widely used approach in current regulatory network inference studies. Given the inherent sparsity of gene regulatory networks, the accuracy (ACC) metric is not suitable for network evaluation. as it may overestimate performance due to the large number of true negatives in sparse networks. Therefore, we opted to evaluate CDD using the area under the curve (AUC) and the area under the precision‐recall curve (AUPR), presenting network visualizations for select networks. We also compared some other latest approaches in Section S2 (Supporting Information).

For the algorithm comparison, we utilized the synthetic and real datasets, including DREAM4,^[^ ^19^ ^]^ Hela,^[^ ^20^ ^]^ and IRMA^[^ ^3^ ^]^ datasets, widely recognized as benchmark datasets for gene regulatory network inference. CDDs were compared against other inference techniques on these datasets, consistently demonstrating superior performance.

#### 2.2.1 Synthetic Dataset of Gene Regulation Network

**Figure 2:**
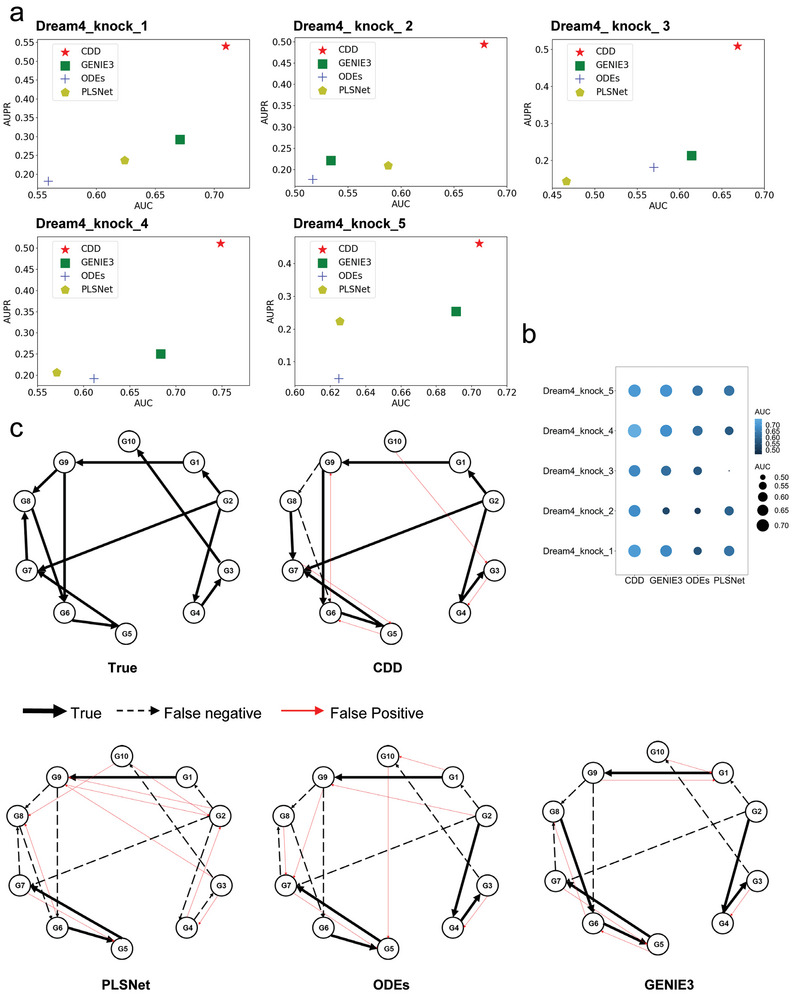
Inferring gene regulatory networks from DREAM4 datasets. Panel (a) represents the AUC and AUPR for inferring the gene regulatory network of the CDD and other 3 methods using DREAM4. Panel (b) represents AUC comparison results between methods five datasets. Panel (c) represent the results of the actual network versus the predicted network drawn from different methods. “True” represents the real network. CDD, GENIE3, PLSNet, and ODES represent the network inferred from CDD, GENIE3, PLSNet, and ODES respectively. The red line indicates what was not predicted, the black solid line indicates what was correct, and the black dashed line indicates what was predicted incorrectly. The sample size of the dream4 dataset here is 10.

We obtained five sub‐datasets from the DREAM4 challenge,^[^ ^21^ ^]^ which include both network structure and gene expression data. We used the gene expression data to infer the gene regulatory network by CDD and other existing methods, and the CDD outperformed other existing methods in both AUC and AUPR (**Figure** **2**). For instance, in the second DREAM sub‐dataset, our method achieved an AUC value of 0.6373, outperforming the optimal value of 0.5878 from PLSNet, which has the best AUC in other methods (Figure 2b). Additionally, our AUPR value of 0.4937 significantly surpassed the optimal value of 0.2209 from GENIE3, which has the best AUPR in other methods (Figure 2a). These two metrics demonstrate the superiority of our method over other approaches. Our algorithm exhibits higher accuracy in network inference, as evidenced by its superior performance in terms of AUC and AUPR metrics when compared to the other three algorithms (Figure 2). Moreover, CDD offers a distinct advantage in practical applications. While other methods generate probabilistic values to indicate network relationships, they lack direct confirmation of these relationships. This poses challenges in determining an appropriate classification threshold based on these values. In contrast, our algorithm directly determines the presence or absence of causal relationships. Unlike other methods that rely on predefined thresholds to match top‐n edges in the actual data network, our algorithm does not require prior knowledge of the number of edges. For the prediction result of the network structure of the DREAM4 size10_knockdown5dataset, the CDD inferred 9 correct edges and 7 incorrect edges compared with the true network, GENIE3 predicted 6 correct edges and 12 incorrect edges, PLSNet predicted 5 correct edges and 14 incorrect edges, and ODEs only predicted 3 correct edges and 18 incorrect edges (Figure 2c). So, our method can infer more correct edges (represented by black solid lines) and fewer incorrect edges (represented by red lines and black dashed lines) (Figure 2c). This underscores the practical effectiveness of our method.

#### 2.2.2 Real Dataset of Gene Regulation Network

**Figure 3:**
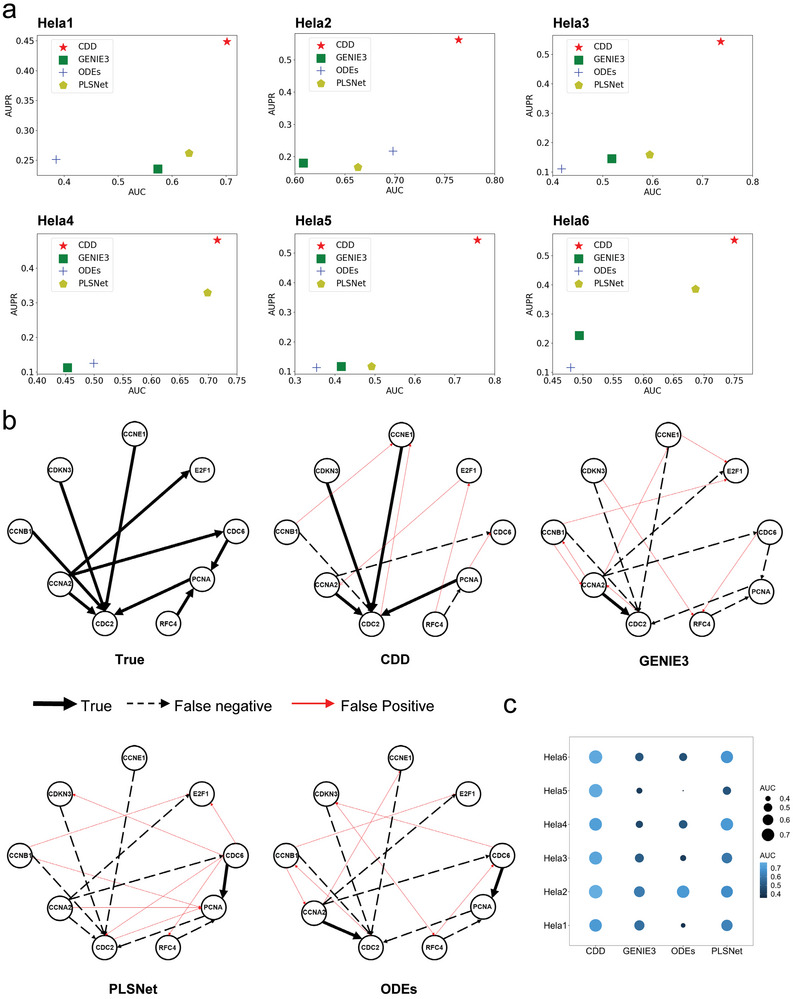
Inferring the gene regulatory networks from Hela datasets. Panel (a) represents AUC and AUROC. Red indicates the results of our method. Panel (b) represents the results of actual versus predicted plots of gene regulations. “True” represents the real network. CDD, GENIE3, PLSNet, and ODES represent the network inferred from CDD, GENIE3, PLSNet, and ODES respectively. The red line indicates what was not predicted (as in the standard), the black solid line indicates what was correct, and the black dashed line indicates what was predicted incorrectly. Panel (c) represents AUC comparison results between methods in six datasets. The sample size of the Hela dataset here is 9.

The Hela dataset,^[^ ^20^ ^]^ a widely used dataset for gene regulatory network inference in cancer cells, consists of six sub‐datasets, each accompanied by its corresponding gene expression data. These datasets provide valuable insights into the regulatory network governing gene relationships. We used the gene expression data to infer the gene regulatory network by CDD and other existing methods. Our method demonstrated significant improvements over the other three approaches, consistently outperforming them in terms of network inference accuracy and effectiveness (**Figure** **3**a).

For instance, in the first sub‐dataset hela1, our method achieved an AUC value of 0.7013, surpassing the optimal methods that attained only 0.6319 from PLSNet which is the best performance method in other methods (Figure 3a). Similarly, our AUPR value of 0.4486 was 18 percentage points higher than the optimal methods' value of 0.2618 from PLSNet (Figure 3a). These superior results were consistently observed across the other Hela datasets (Figure 3a). In terms of actual inference results of the Hela2 dataset, CDD predicted 4 correct edges, and it outperforms the other three methods by inferring more true edges and making fewer incorrect edge judgments (Figure 3b). Importantly, our method does not rely on the number of known true edges, making it more flexible and adaptable to different scenarios. These findings highlight the improved performance and robustness of our method in inferring gene regulatory networks using the Hela dataset. Our algorithm consistently outperforms other approaches, demonstrating higher accuracy and effectiveness in network inference (Figure 3).

**Figure 4:**
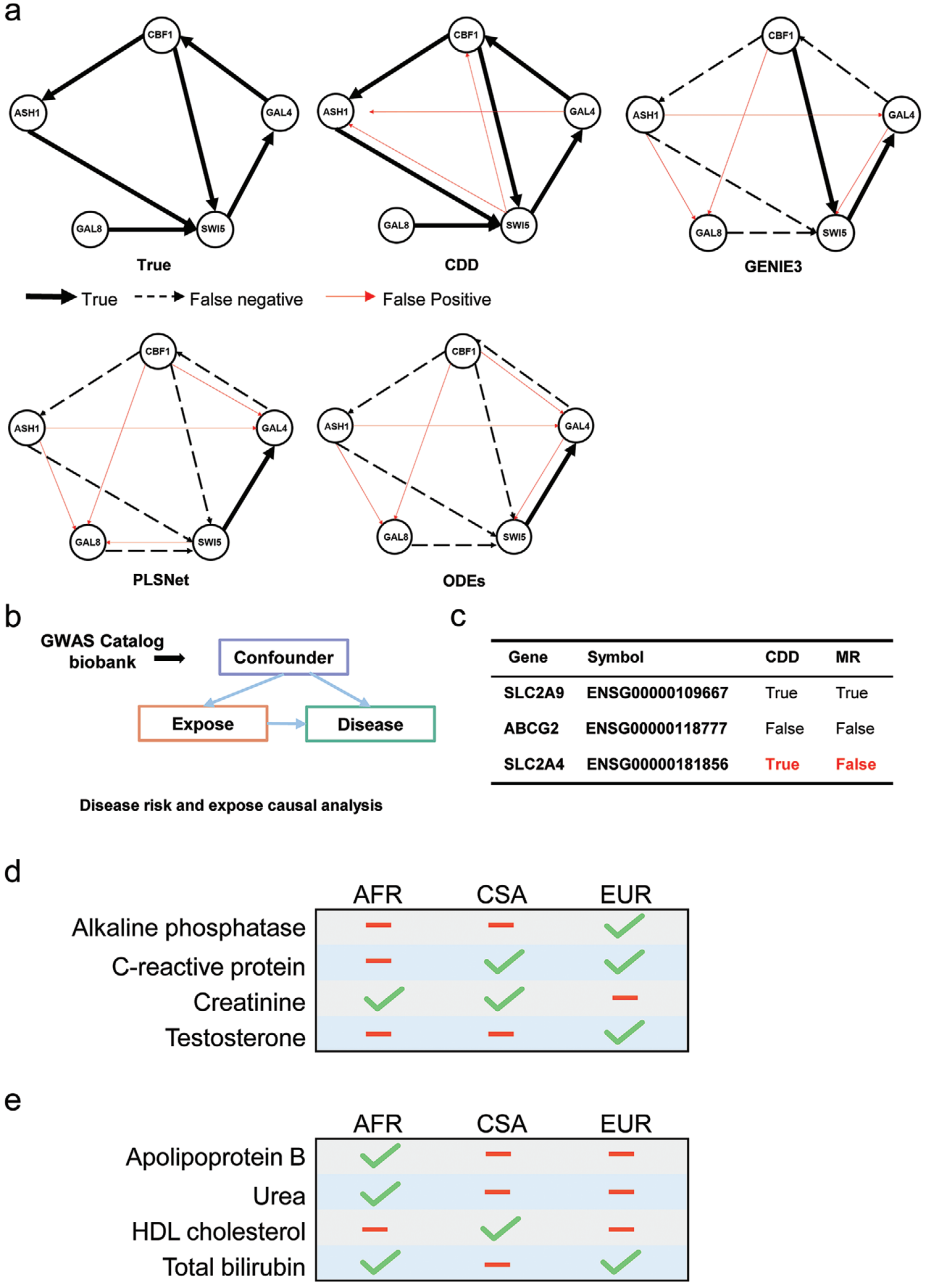
Inferring the gene regulatory networks from the IRMA dataset and kidney dataset. Panel (a) represents the results of the IRMA dataset. Results of actual versus predicted plots of gene regulations. “True” represents the real network. CDD, GENIE3, PLSNet, and ODES represent the network inferred from CDD, GENIE3, PLSNet, and ODES respectively. The red line indicates what was not predicted (as in the standard), the black solid line indicates what was correct, and the black dashed line indicates what was predicted incorrectly. The sample size of the IRMA dataset here is 16. Panel (b) stands for CDD using GWAs data from Catalog and UK Biobank for causal analysis of disease and exposure. Panel (c) represents the kidney dataset results from Mendelian randomization (MR). Panel (d) represents the causal analysis results of prostate cancer and the potential factors in different populations (UK Biobank). Panel (e) indicate the causal relationship between colorectal cancer and potential factors in different populations (UK Biobank).

The IRMA dataset,^[^ ^3^ ^]^ which consists of expression data and standard network structure for five genes in yeast cells, was used to evaluate our proposed method. Notably, CDD demonstrated superior performance, predicting more correct edges and fewer incorrect edges compared to other methods (**Figure** **4**a). In the case of a real network with six directed edges, our method can accurately predict all of the real edges (Figure 4a) and 3 incorrect edges (Figure 4a). ODEs, the best of the other methods, only predicted 2 real edges and 8 incorrect edges (Figure 4a). This highlights the effectiveness of CDD in real‐world datasets.

### 2.3 Causality Inference of Diseases and Phenotypes

A significant aspect of analyzing causal relationships between diseases and phenotypes involves the analysis of Single Nucleotide Polymorphisms (SNPs). SNPs data plays a crucial role in this analysis of genome‐wide association studies. The most commonly used approach for inferring causality from SNP to phenotype is Mendelian randomization,^[^ ^22^ ^]^ which relies on the idea of instrumental variables. However, instrumental variables face challenges, such as identifying suitable instrumental variables and accounting for multifactorial effects (multifactorial Mendelian randomization^[^ ^10^ ^]^ suffers from covariance issues). While Mendelian randomization partially addresses the instrumental variable identification problem, multifactorial effects remain a substantial challenge, often leading to the failure of Mendelian randomization. To address these challenges, we applied the CDD method to SNP datasets and compared its performance with instrumental variable approaches, i.e., Mendelian randomization methods, and nominal significance (NS).

#### 2.3.1 Synthetic Dataset of SNPs Dataset

For the comparison of Mendelian randomization, we utilized the challenge dataset, MRDataChallenge2019 dataset, provided by the 2019 Mendelian Randomization Conference (https://www.mendelianrandomization.org.uk/). This dataset encompasses pooled data on estimated associations between 118 metabolic factors and 150 genetic variants. Metabolite information was quantified using nuclear magnetic resonance (NMR) spectroscopy metabolomics. Additionally, the dataset included information on seven outcomes, age‐related macular degeneration, Alzheimer's disease, type 2 diabetes mellitus, ischemic stroke, aortic stroke, cardiac stroke, and small vessel stroke. To facilitate comparisons, we focused on the first 14 metabolic factors, excluding less‐studied substances. Our primary purpose is to examine the differences between CDD and Mendelian randomization and make comparisons based on these differences. Applying Mendelian randomization and our algorithm to this dataset, we used published articles as a benchmark to evaluate our algorithm against the Mendelian randomization algorithm (Tables S5 and S6, Supporting Information).

**Figure 5:**
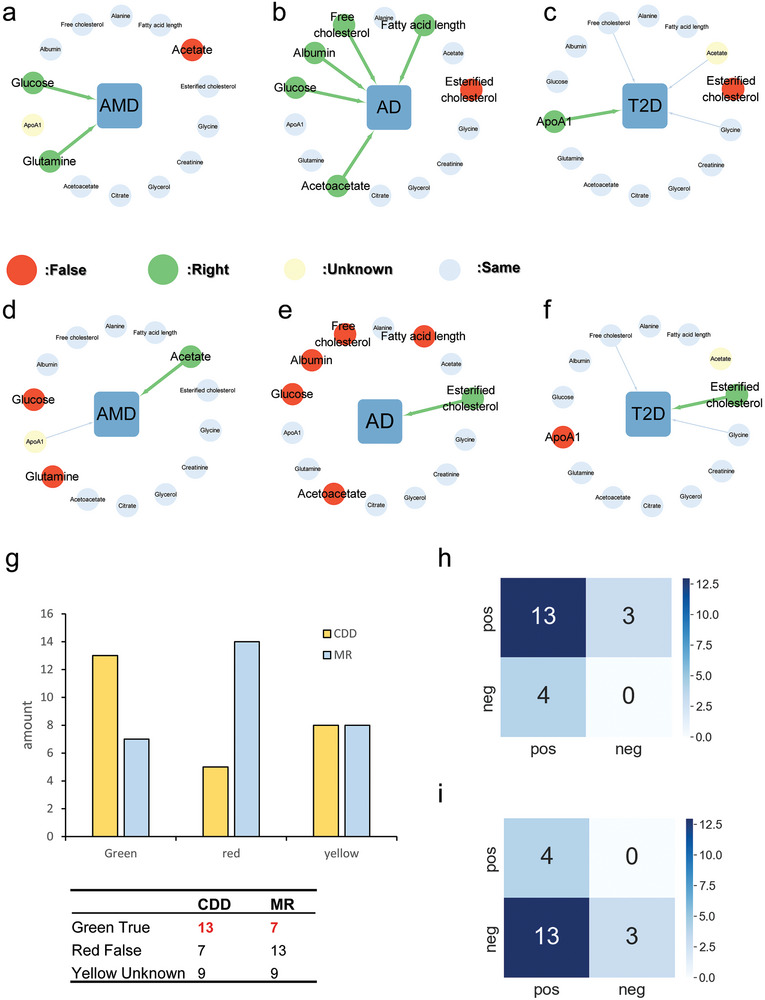
The comparison results on the 2019 challenge dataset in the SNP problem. Here the relationship between phenotype and disease was analyzed by CDD and Mendelian randomization (MR). The middle green indicates the disease, the peripheral gray indicates the consistent results obtained by the two methods, the red indicates the incorrect results, and the green indicates the correct results. Light yellow indicates that there is no reference result yet (i.e., it is not possible to determine whether it is correct or incorrect). Panel (a–c) show the results of CDD, Panel (d–f) show the results of MR. Panel (g) represents the resulting data statistics between CDD and MR. After excluding Yellow Unknown, Panel (h) is the confusion matrix of CDD results and Panel (i) is the confusion matrix of MR Results.

We compared several of these datasets using both our algorithm and Mendelian randomization to infer the relation between metabolic factors and disease and found that the CDD (**Figures** **5**a–c) outperformed the Mendelian randomization (Figures 5d–f). For example, the results show that CDD can predict two metabolic factors (glucose and glutamine) for age‐related macular degeneration (**Figure** **5**a), and the Mendelian randomization can only predict one metabolic factor (acetate) for age‐related macular degeneration (Figure 5d). Existing studies^[^ ^23^, ^24^, ^25^ ^]^ have shown that age‐related macular degeneration can be affected by these factors, i.e., glucose, glutamine, and acetate (Tables S5 and S6, Supporting Information).

Similarly, the CDD can predict five proved metabolic factors (acetoacetate,^[^ ^26^ ^]^ glucose,^[^ ^27^ ^]^ albumin,^[^ ^28^ ^]^ free cholesterol,^[^ ^29^ ^]^ and fatty acid length^[^ ^30^ ^]^) for the Alzheimer's disease (Figure 5b and Table S5, Supporting Information), and the Mendelian randomization can only predict one proved risk factor (Esterified cholesterol^[^ ^31^ ^]^) for the Alzheimer's disease (Figure 5e and Table S6, Supporting Information). For example, the existing publication has proven that the changes in glucose uptake in the cerebral cortex may predict the histopathological diagnosis of Alzheimer's disease.^[^ ^32^ ^]^ plasma exchange with albumin replacement could slow cognitive and functional decline in Alzheimer's disease.^[^ ^28^ ^]^ Among the differential results, our method correctly identified 13 cases and made 7 incorrect judgments (Figures 5e,f). In contrast, Mendelian randomization achieved correct judgments in 7 cases but made 13 incorrect judgments (Figure 5g). There are 9 cases not find the corresponding relationships with the diseases from the references, so their relationships are unclear and could not be conclusively determined (Figure 5g). Unclear often means that there are not many studies on the metabolic factor at present, and it cannot be judged whether the metabolic factor does not affect the disease. The incorrect judgment is based on determining that the relationship between the metabolic factor and the disease is already supported by the literature, or that the study of the substance is rich, but there is no study related to the disease. It is challenging to ascertain whether the absence of research on the relationship between metabolic factors and diseases is due to a lack of investigation or an actual absence of a relationship. The unclear results were excluded, and the remaining results were analyzed by like confusion matrix (Figures 5h,i), in which the number of True Positives and True Negatives of the CDD method was significantly better than that of the MR Method. Importantly, CDD provided more reliable results than Mendelian randomization, yielding a higher number of accurate predictions. This highlights the effectiveness and robustness of our approach in inferring relationships between metabolite factors and disease outcomes.

#### 2.3.2 Identifying Genes Causally Associated with Kidney Disease Using SNPs

We also conducted a comparison using datasets related to kidney disease.^[^ ^33^ ^]^ Specifically, we examined an article that investigated the involvement of glut9 (Human Glucose Transporter‐Like Protein‐9) in chronic kidney disease using Mendelian randomization (Figure 4c). Glut9 is known to encode a member of the SLC2A facilitative glucose transporter family, which plays a crucial role in maintaining glucose homeostasis. Remarkably, the CDD was able to replicate the same result, providing validation for the role of glut9 in kidney disease (Figure 4c). Furthermore, we expanded the CDD to explore the relationship between glut4 and chronic kidney disease using Mendelian randomization. While Mendelian randomization failed to establish a conclusive relationship between glut4 and the disease. In contrast, our algorithm successfully identified a significant relationship between glut4 and chronic kidney disease, aligning with the findings reported in the published article.^[^ ^34^ ^]^ The study suggests that the downregulation of glut4 in skeletal muscle may be associated with insulin resistance in chronic kidney disease.^[^ ^34^ ^]^ The confirmed relationship between glut4 and chronic kidney disease further supports the effectiveness and reliability of our algorithm in uncovering meaningful associations.

#### 2.3.3 Lipidomic Data in Relation to Coronary Artery Disease

**Figure 6:**
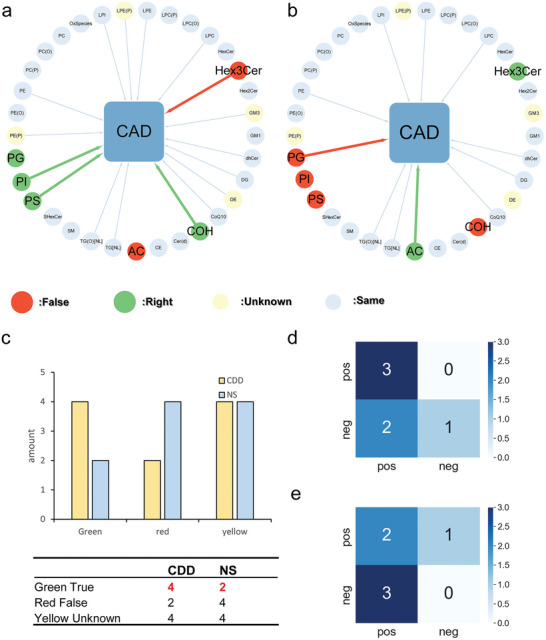
The comparison results of the causal relations between the whole lipid group and coronary.artery disease (CAD). Panel (a) represents the results inferred from CDD, and Panel (b) represents the results inferred from nominal significance (NS). The middle green indicates the disease, the peripheral gray indicates the consistent results obtained by the two methods, the red indicates the incorrect results, and the green indicates the correct results. Light yellow indicates that there is no reference result yet (i.e., it is not possible to determine whether it is correct or incorrect). Panel (c) represents the resulting contrast histogram comparison between CDD and NS. After excluding Yellow Unknown, Panel (d) is the confusion matrix of CDD results, and Panel (e) is the confusion matrix of NS Results.

The data we utilized in our study was sourced from an existing article,^[^ ^35^ ^]^ which presented whole lipidome data comprising 33 lipid major classes and 596 lipid minor classes. The population under investigation in the article was European, and the data was in the form of SNP data. Lipids are known to have close associations with coronary artery disease, but identifying the specific lipid substances that play a role is crucial. To evaluate the performance of our method, we conducted a comparison with the approach employed in the original paper named nominal significance (NS).^[^ ^35^ ^]^ The NS is a method based on a significance test. Specifically, we focused on analyzing the 33 lipid major classes (Table S7, Supporting Information) and compared the results obtained from the CDD (**Figure** **6**a) with the method reported in the original article (Figure 6b). For instance, free cholesterol (COH) has been proven as a risk indicator in patients with angiographically documented coronary artery disease.^[^ ^36^ ^]^ Upon conducting the comparative analysis, we found that the CDD achieved 4 correct judgments, which surpasses the 2 correct judgments reported in NS (Figure 6c and Table S7, Supporting Information). Using confusion matrix analysis (Figures 6d,e), The number of True Positives and True Negatives of the CDD method was significantly better than that of the NS Method. This suggests that our algorithm outperforms other algorithms, providing more effective results and potentially identifying lipid factors that may be associated with coronary artery disease. These findings emphasize the superior performance of our algorithm and its ability to uncover potential lipid factors that may play a role in coronary artery disease. By leveraging our method, we can obtain improved insights into the relationship between lipids and coronary artery disease.

We can demonstrate from the results that our method is versatile, and it can achieve more accurate results compared to other methods across different biology fields. In addition, we also have made some achievements in the problem of causal inference in the economic field (Section S2 and Figure S4, Supporting Information).

The public references used as comparison standards are listed in Tables S5–S7 (Supporting Information), providing a comprehensive overview of the sources we consulted.

### 2.4 Application in UK Biobank Database

We utilized genome‐wide association study (GWAS) data from the UK Biobank (https://www.ukbiobank.ac.uk/) to investigate the causal relationships between diseases and various factors across different ethnic groups. We studied the potential causality from the underlying factors to four diseases, including prostate cancer, colorectal cancer, lipoma of skin and subcutaneous tissue (Section S2, Supporting Information), and disorders of lipoid metabolism (Section S2, Supporting Information).

#### 2.4.1 The Potential Factors for Prostate Cancer

We identified 27 potential disease factors and 3 racial populations (Central/South Asian, African, and European) stored in the UK Biobank database related to prostate cancer. We used the CDD method to calculate the causality of the disease factors to prostate cancer and estimate the potential impact of the disease factors on prostate cancer for the 3 populations. Then, we found most of the disease factors can be related to prostate cancer, but, interestingly, different disease factors can be related to prostate cancer in different race populations (Figure 4d and Table S8, Supporting Information). For example, the alkaline phosphatase is an isoenzyme located on the outer layer of the cell membrane,^[^ ^37^ ^]^ and testosterone is a major sex hormone to the development of male growth and masculine characteristics.^[^ ^38^ ^]^ In our results, alkaline phosphatase and testosterone were found to have no causal effect on prostate cancer outcomes in African and Central/South Asian populations, while they did show a causal effect in European populations. Alkaline phosphatase is related to acyl carrier protein (ACPP) and nucleic acids phylogenetic profiling (NAPP), and testosterone is associated with hydroxysteroid 17‐beta dehydrogenase (HSD17). Previous prostate cancer studies have reported that ACPP, NAPP, and HSD17 are specific genes for prostate cancer in European populations compared to African, Asian, and Hispanic populations.^[^ ^39^ ^]^ The findings also indicate that the factors that affect prostate cancer in the European population, are consistent with the result that prostate cancer may be associated with genetic factors related to ethnicity.^[^ ^40^ ^]^ According to CDD algorithm results, alkaline phosphatase, testosterone, and glucose exhibited causal effects for prostate cancer only in the European population (Figure 4d and Table S8, Supporting Information), it is consistent that prostate cancer may be associated with genetic factors related to ethnicity.

#### 2.4.2 The Potential Factors for Colorectal Cancer

We used CDD to predict causality and estimate the potential impact of the disease factors on colorectal cancer for different populations (Figure 4e and Table S9, Supporting Information). The potential disease factors and three racial populations are the same as those for prostate cancer, comprising 27 disease factors and three populations. We found that these four substances from the 27 disease factors have no causal relationship to colorectal cancer in the 3 populations (Table S9, Supporting Information). Additionally, the apolipoprotein B and urea were found to have no causal effect on colorectal cancer outcomes in European and Central/South Asian populations, while they did show a causal effect in African populations (Figure 4e and Table S9, Supporting Information). Apolipoprotein B is the primary apolipoprotein and is the carrier for other lipids,^[^ ^41^ ^]^ and urea is the main nitrogenous end product of protein metabolic decomposition.^[^ ^42^ ^]^ However, protein and lipid intake in Africa is generally lower than that in Eurasia, so we speculate that insufficient and uniquely nutrient intake may be one of the factors for colorectal cancer to be affected by apolipoprotein B and urea in African populations.

## 3 Discussion

Our CDD method has shown exceptional performance in addressing the challenges of gene regulatory network inference and inferring disease‐phenotype relationships. Our approach has outperformed widely used methods in their respective domains, providing compelling evidence for the effectiveness of the CDD framework.

To improve the identifiability of causality, we introduce H0 and H1 assumptions, which strengthen the rigor of our approach. We are also the pioneers in combining diffusion models with do‐calculus, which sets our method apart from others. Unlike some approaches that rely on additional information, such as transcription factor data, the CDD does not require such information.

A key strength of CDD is its explicit consideration of interventional causality. This distinguishes our approach from others (e.g., association study or observational causality) and provides a definitive characterization of causal relationships, avoiding ambiguous values and unclear thresholds. This feature is highly valuable in the biological field, as it offers researchers a clearer reference point and guides their investigations.

Importantly, our models have successfully identified potential causal factors associated with kidney disease and other diseases, which could not be determined using Mendelian randomization alone. Some of these findings have been validated in the literature, further confirming the credibility and reliability of our approach. In particular, the causal analysis between various diseases and the potential factors in different populations from the UK Biobank database further validated the effectiveness of CDD. The remaining discoveries provide additional insights for researchers in the biological field, expanding the knowledge base and stimulating further exploration. Crucially, our approach leverages the principle of causality, allowing for its universal application across diverse data domains.

By combining the diffusion model, the challenge or difficulty that do‐calculus suffers from can be resolved. The integration of these two methods not only maintains the theoretical consistency of causality inference but also extends its applicability to a wider variety of data types. For example, GENIE3, based on random forests, actually remains a correlation‐based method without incorporating causal analysis. In contrast, our approach, through the integration of the do‐calculus and diffusion model, theoretically ensures causal relationships between variables in the sense of intervention. Furthermore, while methods like Granger causality and Mendelian randomization (an instrumental variable approach) are grounded in (non‐interventional) causal theory, each of them is limited by other external factors. Granger causality is restricted to time series data, and Mendelian randomization requires the identification of suitable instrumental variables, such as specific Single Nucleotide Polymorphisms (SNPs). However, our method combining the diffusion model with do‐calculus can analyze both time series data and instrumental variables. By comparison with existing methods for causality inference, the results also demonstrate advantages over other existing methods. Additionally, we have shown that do‐calculus cannot simply be combined with standard generative models, such as autoencoders, to infer causality (Section S1, Supporting Information).

For the case where the dependent variable *x* is not a single variable, but a vector. The CDD also has certain application scenarios, in which the do operation is not carried out on scalar *x*, but on multiple *x* at one time. In this case, we can view the multiple *x* as a whole *X*.

## 4 Materials and Methods

### 4.1 H0 and H1 Framework

By extending the Granger causality framework for time series data, we developed H0 and H1 assumptions on non‐time series data,^[^ ^14^ ^]^ which enables the assessment of causal relationships between targets on high‐dimensional data. Specifically, based on the initial correlation network, H0 assumption (i.e., no causal relationship or no edge from gene *x* to *y*) and H1 assumption (i.e., causal relationship or an edge from gene *x* to *y*) were applied to each pair of two variables from *x* to *y* or an initial edge from gene *x* to *y* in a network, where *x* ∈ *R* and *y* ∈ *R* are 1D variables (real numbers), and *R* means the set of real number. Different regression equations (i.e., ANM) were obtained by using gene expression data based on different assumptions (using the training dataset). As shown in Figure 1b, in the H0 hypothesis, regression fitting of the outcome variable *y* is performed using other variables Z = (*z*_1_,*z*_2_,_⋅⋅⋅_, *z*_*n* − 2_) ∈ *R* ^*n* − 2^ that do not include the dependent variable *x* by regression function *f*_*H*0_. In H1 hypothesis, the dependent variable *x* and other variables *Z* were used to fit the outcome variable *y* by regression function *f*_*H*1_. Note that there are *n* observed variables (including *x* and *y*). The specific regression formula is as follows:

(1) $$\text{H0:}\;\;y=f_{H0}\;\left(z_{1},z_{2},\text{…},z_{n-2}\right)+\hat{\varepsilon }$$

(2) $$\text{H1:}\;\;y=f_{H1}\left(x,z_{1},z_{2},\text{…},z_{n-2}\right)+\varepsilon$$

where *y* represents the outcome variable and *x* represents the dependent variable. *Z* are other (*n*‐2) variables (a vector) that do not contain variables *x* and *y*. ε and $\hat{\varepsilon }$ represent the noises or errors. Note that we divide the data into a training dataset and a testing dataset, and the dataset used in this training stage for H0 and H1 is the training dataset, which is used to train a neural network as the nonlinear functions *f*_*H*0_ and *f*_*H*1_. In the next testing stage, we will test the assumptions by using the testing dataset.

According to the regression Equations (1) and (2) under such two assumptions learned from the training dataset, the errors under the two different assumptions were obtained by bringing them into the remaining testing dataset. In this testing stage, by comparing the errors between any two variables (using the testing dataset), the causal H1 or non‐causal H0 relation from *x* to *y* can be judged. Testing H0 and H1 for each pair of variables or any initial edge from *x* to *y* among all observed variables, their whole causal network can be constructed in such a way. Meanwhile, the influence of other variables *Z* on *y* is also excluded.

It should be noted that in this method, to ensure the comparability of the fitting error, the data needs to be divided into a training set and a testing set. The data of the training set is used for the fitting of the neural network, and the data of the testing set is used to obtain the fitting errors $\hat{\varepsilon }$ and ε. The main way to divide the data set was a *K*‐fold cross‐test. Constructing Equations (1) and (2) by a neural network from the data is simply an association study, rather than causal inference, and it can be viewed as an extension of the Granger causal inference from time series data to cross‐sectional (non‐time series) data. To reliably test the assumptions H0 and H1 causally, next we show our new strategy in this H0 and H1 framework, i.e., diffusion do‐calculus (CDD), which uses the diffusion model to intervene variables, and then determines the causal relationship from any variable *x* to *y* through the do‐calculus intervention. Finally, by comparing the generated results with the real results, we can judge whether the assumptions H0 and H1 are satisfied causally to reconstruct the causal network.

### 4.2 Causal Diffusion Do‐Calculus Model (CDD) with Deep Learning

#### 4.2.1 Diffusion Model

The diffusion model is an end‐to‐end generative model that consists of two main components, the forward process (noising) and the reverse process (denoising). It has found extensive applications in tasks such as image and text generation, showcasing its impressive generation capabilities. In the context of this strategy, the diffusion model is utilized based on the three key aspects, generation performance, end‐to‐end modeling, and forward diffusion process, which allow us to generate the hypothesized relationships in H0 and H1, perform specific operations on input variables, and further alleviate the noise effect.^[^ ^43^ ^]^

The diffusion model is divided into forward and backward diffusion processes, both of which are Markov processes. The forward process is the noise‐adding process, which diffuses the original data distribution into noise. In Figure 1b, we assumed variables D = (*x*, *Z*, *y*)  ∈ *R^n^* as the data domain, and their corresponding *H*  =  (*h_x_*, *h_Z_*, *h_y_*) ∈ *R^n^* are hidden layer variables. The *t* from 1 to T represents the diffusion step in the diffusion model, and we focus on the relationship from variable *x* to *y*. And then we get the following forward and backward processes.

The forward process:

(3) $$P\;\;\;\;\left(h_{x}|D\right)=\prod_{t=1}^{T}p_{\theta_{1}}\left(D_{t}|D_{t-1}\right)\;\;\;$$

The backward process:

(4) $$P\;\;\;\;\left(D|h_{x}\right)=\prod_{t=1}^{T}q_{\theta_{2}}\left(D_{t-1}|D_{t}\right)\;$$

where θ_1_ is the forward process parameter artificially given, while θ_2_ is the backward process parameter that needs to be learned. Through training a diffusion model, our objective is to capture a generative relationship for variable *y*. Therefore, we can obtain a relationship denoted as:

(5) $$y=f_{B}\;\;\left(h_{x},h_{y},h_{Z}\right)=f_{B}\;\left(f_{F}\left(x\right),f_{F}\left(y\right),f_{F}(Z\right))$$

where *f_F_* represents the forward process mapping from input data to the changes in the hidden layer (e.g., from *x* to *h_x_*, or from *y* to *h_y_*), and *f_B_* represents the backward process mapping from the entire hidden layer to *y*.

It is important to note that in this context, the forward process can be predetermined and fixed. This means that the forward process can be generated through human manipulation or design. On the other hand, the reverse/backward process requires network learning to generate the mapping function *f_B_* allowing us to reconstruct the original input variables from the hidden layer. Assume that now we have obtained the generative relationship for variable *y* based on the assumptions set forth in H0 and H1, but it remains unknown whether *x* is a dependent variable within this generative relationship. The next step is to determine whether the hypothesis H0 or H1 is satisfied. To assess these assumptions, we can compare the generated results from the diffusion model with the actual observed results. By analyzing the consistency between the generated and real data, we can evaluate or test H0 and H1. This analysis will enable us to make informed judgments about the causal relationship from *x* to *y* and determine which hypothesis is valid or false.

#### 4.2.2 Do‐Calculus on the Hidden Layer

Do‐calculus is an intervention based on the causal theory proposed by Pearl,^[^ ^17^ ^]^ through which we can judge whether or not there is a causal relation from one variable to another (details in Section S1, Supporting Information). In this step, by taking advantage of the intervention operation in the testing dataset, the implementation of the intervention operation is borrowed from OrphicX^[^ ^15^ ^]^. Instead of intervention on the original variables that are difficult to implement, we carry out intervention operations on the hidden layer variables that can not only be implemented but also reconstruct original data. We aim to find the causal relation from *x* to *y* by interfering with *h_x_* (hidden variable of *x*) to see if it affects *y*. Theoretically, such an intervention can be achieved by the do‐calculus framework (Figure 1b). In other words, we perform the do operation on *h_x_* to determine whether or not *x* has a causal effect on *y*. The formula can be expressed as follows:

(6) $$P\;\;\;\;\left(y|do\left(h_{x}\right)\right)=\sum_{x}P\left(y|h_{x},x\right)P\left(x\right)$$

The computational solving process originates from OrphicX,^[^ ^15^ ^]^ with a detailed explanation provided in Section S1 (Supporting Information).

However, common intervention operations often cannot be directly applied to original variables, e.g. we cannot directly change genetic data because the relationship of replication between genes leads to the backdoor criterion^[^ ^17^ ^]^ being often not met. Hence, we performed the do intervention operation on the hidden layer variable instead. The hidden layer is only related to the input data (Figure S1, Supporting Information), thus the backdoor criterion is met between the input data *x* from the hidden layer *h_x_* to the output target *y*.

### 4.3 Testing H0 and H1 Assumptions

The diffusion model was trained on the training dataset in the previous step. Subsequently, the diffusion model was used to perform operations on the variable *x* in the testing dataset. The next step involves assessing the difference between the estimated results ${\hat{y}}_{H1}$ and the actual results *y*, to determine if there were significant differences (Figure 1b).

To account for noise in the ANM model, a second intervention operation was introduced. In this operation, the initial expression of *x* was randomly sampled from Gaussian noise, resulting in a new set of estimated results denoted as ${\hat{y}}_{H0}$. As a result, the judgment of significant differences can be transformed as follows, if the distance/difference between ${\hat{y}}_{H1}$ and the true value *y* is significantly smaller than the distance between ${\hat{y}}_{H0}$ and the true value y, then the H1 hypothesis can be considered accepted. Conversely, if the distance between ${\hat{y}}_{H1}$ and the true value *y* is larger than the distance between ${\hat{y}}_{H0}$ and the true value *y*, then the H0 hypothesis can be accepted.

To measure the distance, metrics such as Hierarchical Image Segmentation and Classification (HISC), Kullback–Leibler (KL), or Mean Squared Error (MSE) can be employed. To compare the error sizes under the two assumptions, if the error of the H0 hypothesis is smaller than that of the H1 hypothesis, it suggests that *x* is not the dependent variable of *y*. In other words, *x* does not have a causal regulatory effect on *y*. Conversely, if the error of the H1 hypothesis is smaller than that of H0, it is assumed that *x* does have a causal regulatory effect on *y*.

In this way, we can judge whether there is causality for each pair from *x* and *y*, and finally obtain a causal network. The algorithm and pseudo‐code of our CDD are shown in **Table** **1**. Based on our CDD algorithm for the causal inference, we can theoretically derive the following result/Proposition.

**Table 1:** Algorithm and pseudo‐code of causal diffusion do‐calculus model (CDD).

1,	Function Output, *x* causes *y* or not. Input, *x_train_*, *y_train_*, *Z_train_*, *x_test_*, *y_test_*, *Z_test_*.
2,	Train diffusion model,
3,	$q_{\theta_{1}}$ ←Artificial given
4,	$q_{\theta_{2}}$ ←$\;\prod q_{\theta_{2}}\left(x_{train},\;y_{train},\;Z_{train}|H\right)$
5,	Obtain generation function, *Y* = *f*_B_ (*h_x_*,*h_y_*,*h_Z_*)
6,	Do‐calculus,
7,	*x*_H1_, *x*_H0_ ← do sampling from *x_test_*, random noise
8,	*h*_*x*1_ ← $q_{\theta_{1}}\left(x_{H1}\right),\;h_{x0}$ ← $q_{\theta_{1}}\left(x_{H0}\right)$
9,	${\hat{y}}_{H1}$ ← *f*_B_(*do*(*h*_*x*1_),*h_y_*,*h_Z_*), ${\hat{y}}_{H0}$ ← *f*_B_(*do*(*h*_*x*0_),*h_y_*,*h_Z_*)
10,	Accept H0 or H1,
11,	If $distance\left({\hat{y}}_{H1},y_{test}\right)>distance\left({\hat{y}}_{H0},y_{test}\right)$
12,	Return, Accept H0, *x* does not cause *y*.
13,	Else if $distance\left({\hat{y}}_{H1},y_{test}\right)<distance\left({\hat{y}}_{H0},y_{test}\right)$
14,	Return, Accept H1, *x* causes *y*.

### 4.4 Statistical Analysis

#### 4.4.1 K‐Fold Cross‐Validation

For the gene regulatory network problem, we applied 3‐fold and 5‐fold depending on the dataset size, while for SNP data, we used the commonly applied 5‐fold.

#### 4.4.2 Evaluation Metrics

The evaluation metrics used in the main text include the Area Under Curve (AUC), and the Area Under the Precision‐Recall Curve (AUPRC). To measure the distance in the main text, metrics such as Hierarchical Image Segmentation and Classification (HISC), Kullback–Leibler (KL), or Mean Squared Error (MSE) are employed.

### 4.5 Code Availability

We showed the code on https://github.com/jc1999123/CDD.

## Conflict of Interest

The authors declare no conflict of interest.

## Supporting information



Supporting Information

## Data Availability

The data that support the findings of this study are openly available in https, //www.mendelianrandomization.org.uk/;https, //www.ukbiobank.ac.uk/ at jc1999123/CDD, causal diffusion do‐calculus analysis (github.com), reference number 19. These data were derived from the following resources available in the public domain, https, //www.mendelianrandomization.org.uk/; https, //www.ukbiobank.ac.uk/; https, //github.com/jc1999123/CDD.
