# Supplementary material for: Reconstructing Molecular Networks by Causal Diffusion Do‐Calculus Analysis with Deep Learning
Source: Adv Sci (Weinh). 2024 Oct 23;11(46):2409170. doi: 10.1002/advs.202409170 (PMC11633463; doi:10.1002/advs.202409170)
Supplement: Supplementary file 1 — Supporting Information [file ADVS-11-2409170-s001.docx]

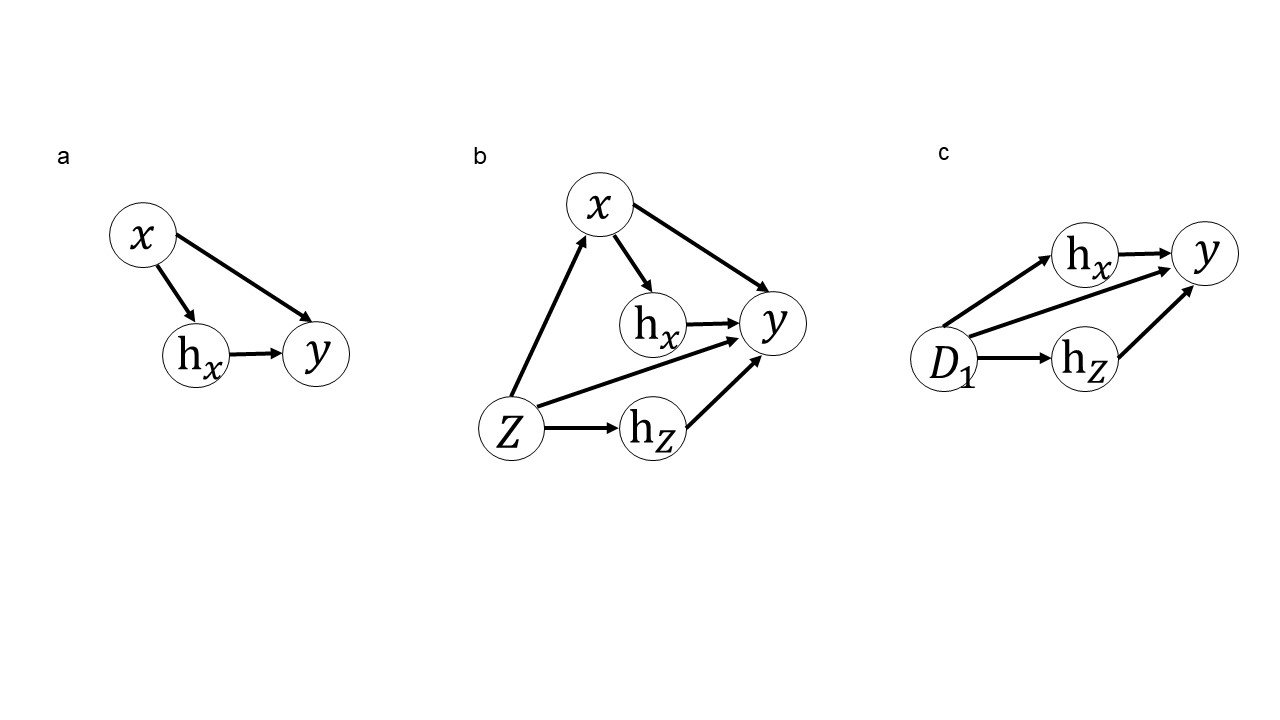


Figure S1. Hidden layer causal diagram.

(a) The causal diagram between $x,y and h_{x}$.

(b) The causal diagram between $x,y ,Z and h_{x}$. (c) The causal diagram between $D,y and h_{x}$.


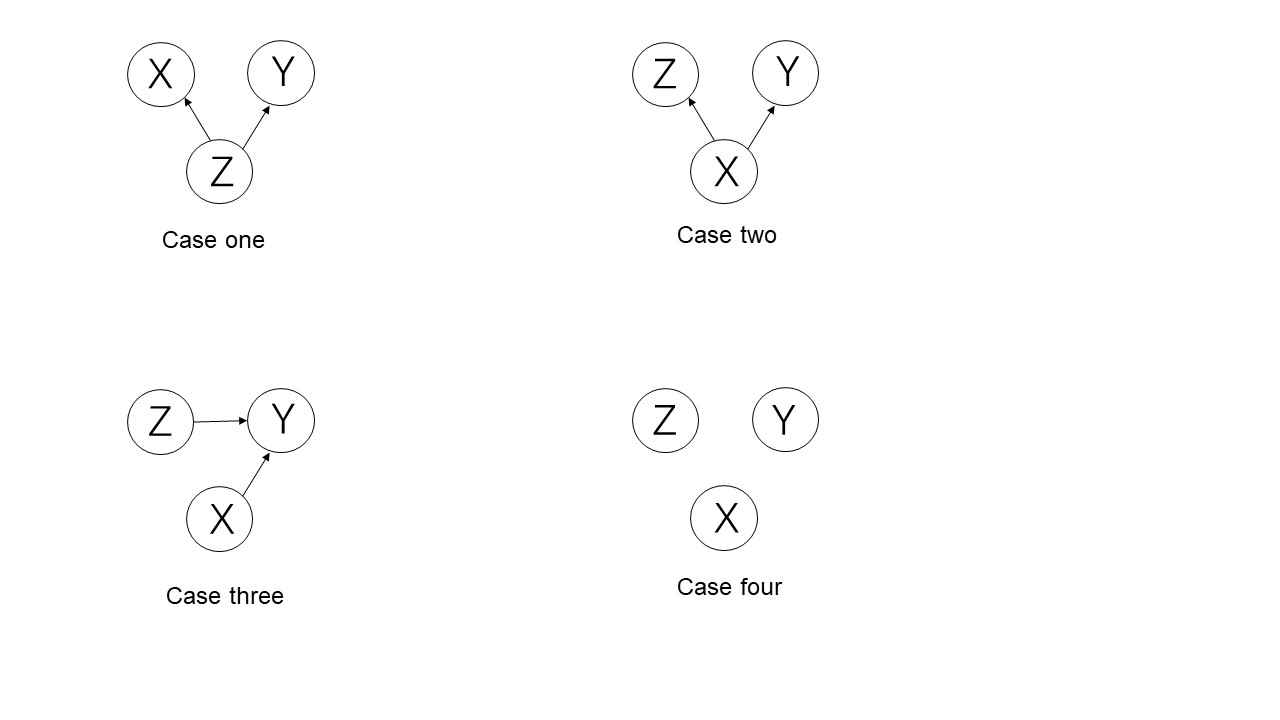


Figure S2. Four causal case

Case one: $X$ has no influence on $Y$, while $Z$ is another variable that has influence on $Y.$

Case two: $X$ has an impact on $Y$, while$Z$ is not affected by other variables.

Case three: $X$ has an impact on $Y$, and $Z$ is other variables affecting $Y.$

Case four: $X$ has no influence on$Y$, and $Z$ is other variables have no influence on $Y.$


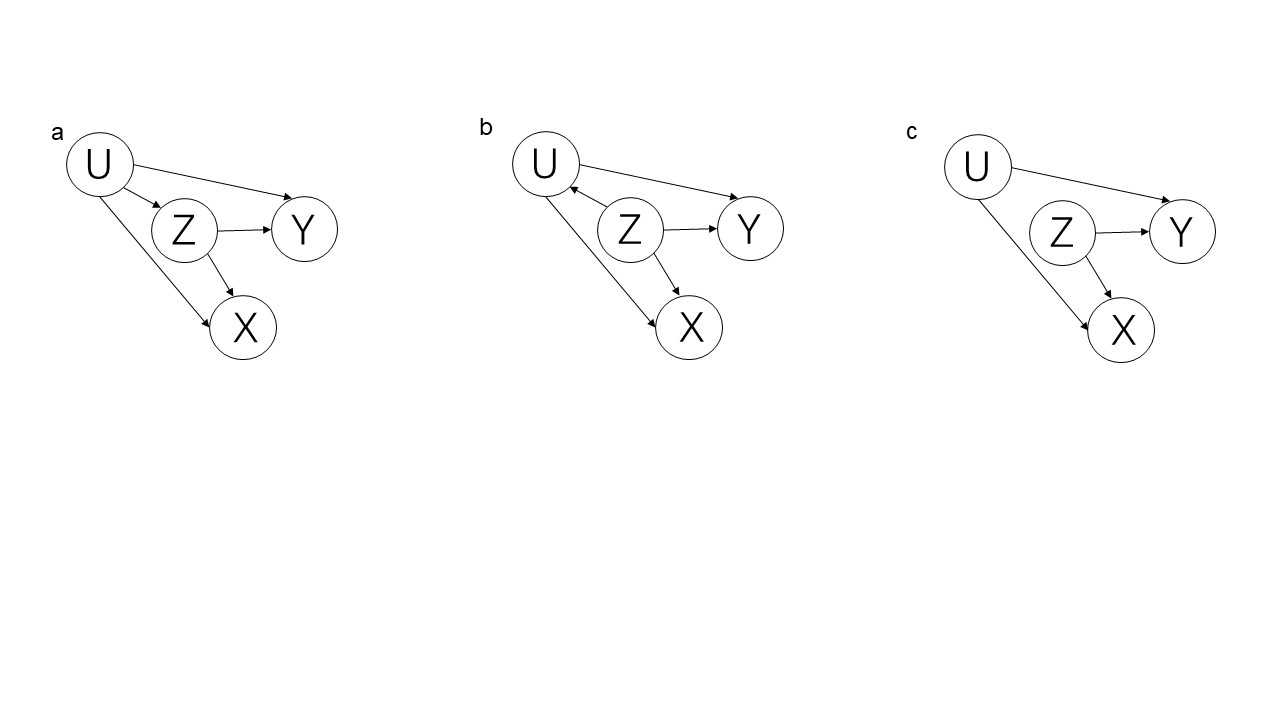
Figure S3. Confounding factors causal diagram.

(a) and (b) represent confounding factors U have causal effect on Z.

(c) represents confounding factors U have no causal effect on Z.

**
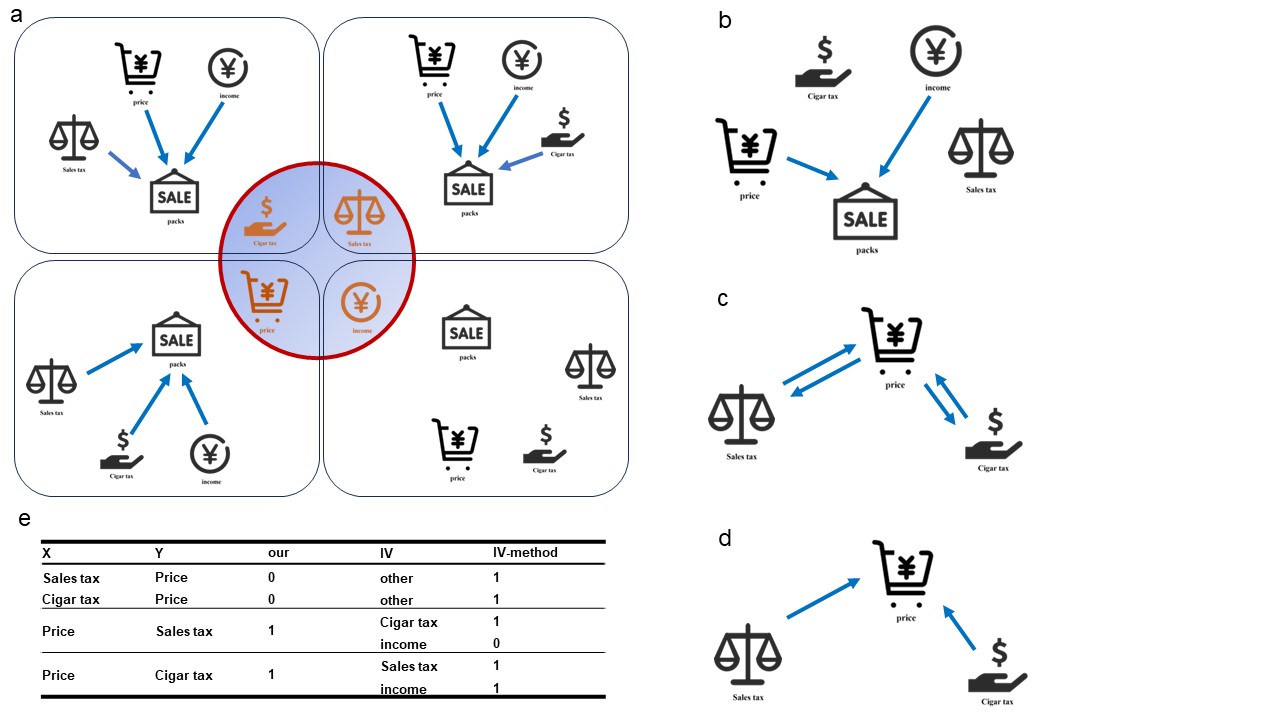
**Figure S4. Cigarette dataset results.

(a) represents the instrumental variable method. The instrumental variables are in the red circles,

(b) and (d) represent CDD results, (c) represents instrumental method result,

(e) a table shows the statistical results for the cigarette dataset.


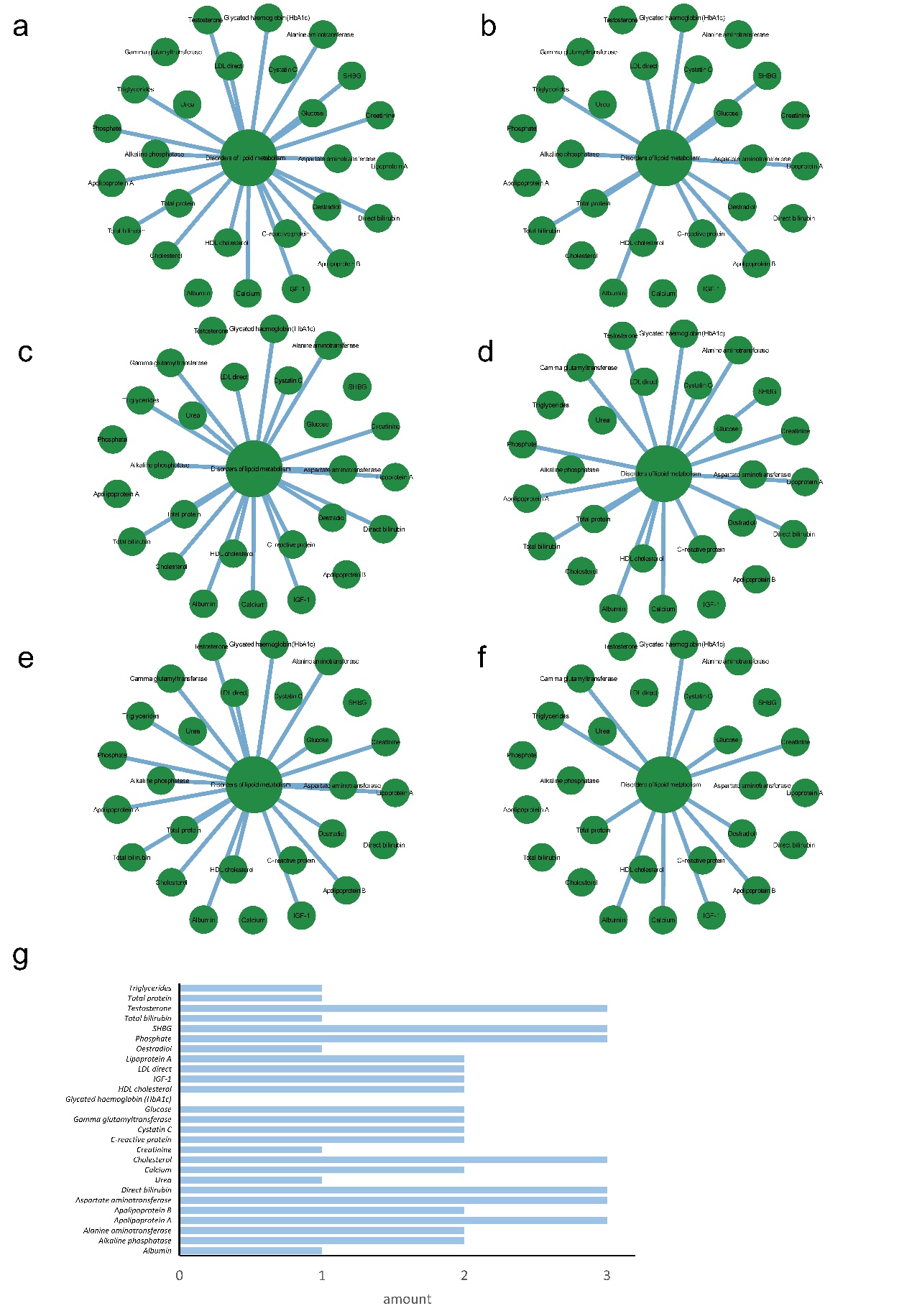


Figure S5. disorders of lipoid metabolism in six different ethnic groups results.

The figure shows the causal relationship between the potential factors and disorders of lipoid metabolism in six populations, (a) – (f) indicating African, Central/South Asian, East Asian, Central Asian, American, and European races, respectively. Edges indicates that the potential factors has a causal effect on disorders of lipoid metabolism. (g) indicates the number of differences in the results of the potential factors among different races.


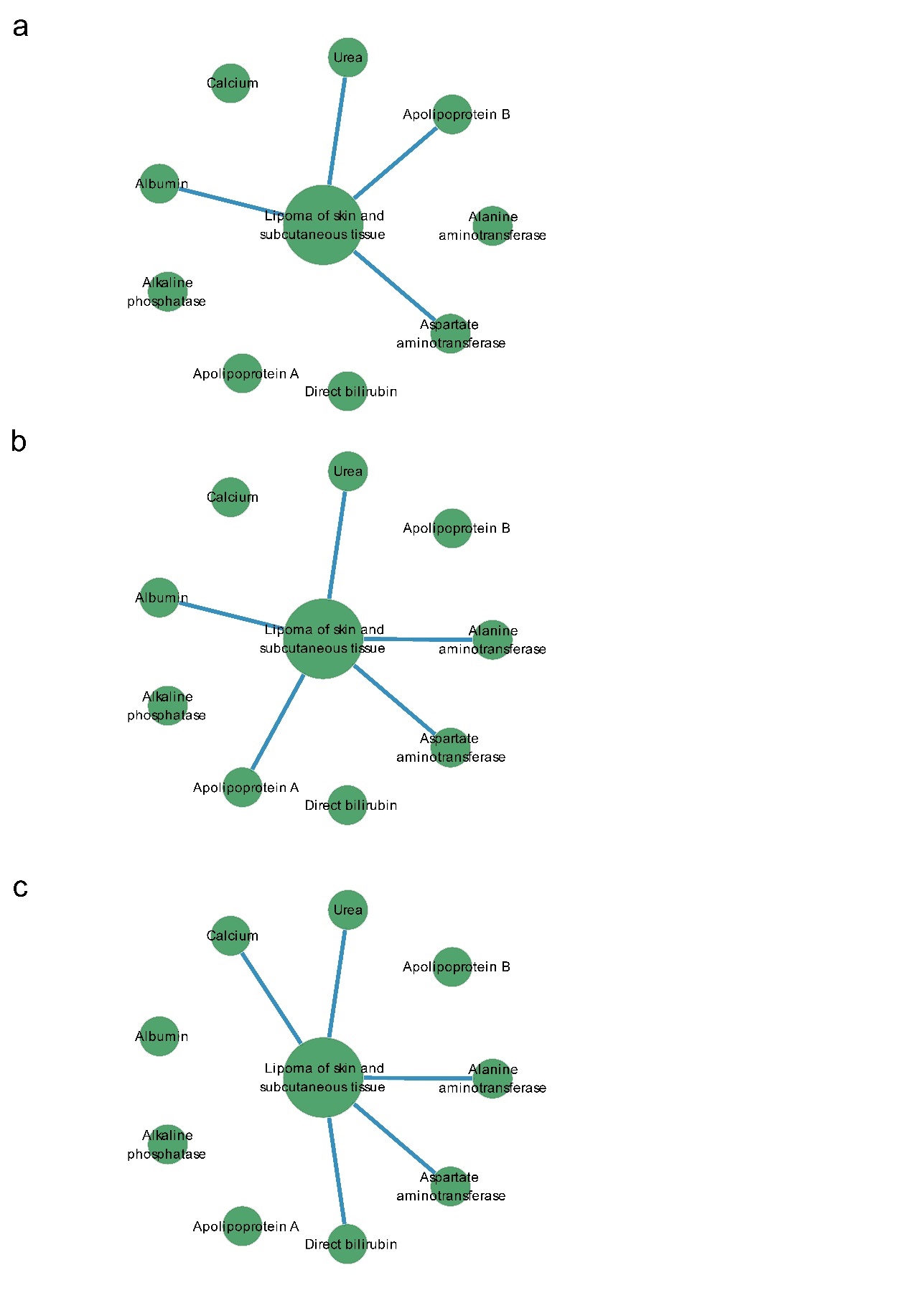


Figure S6. Lipoma of skin and subcutaneous tissue in three different ethnic groups results.

The figure shows the causal relationship between the potential factors and disorders of lipoid metabolism in three populations, (a) – (c) indicating African, Central/South Asian and European races, respectively.


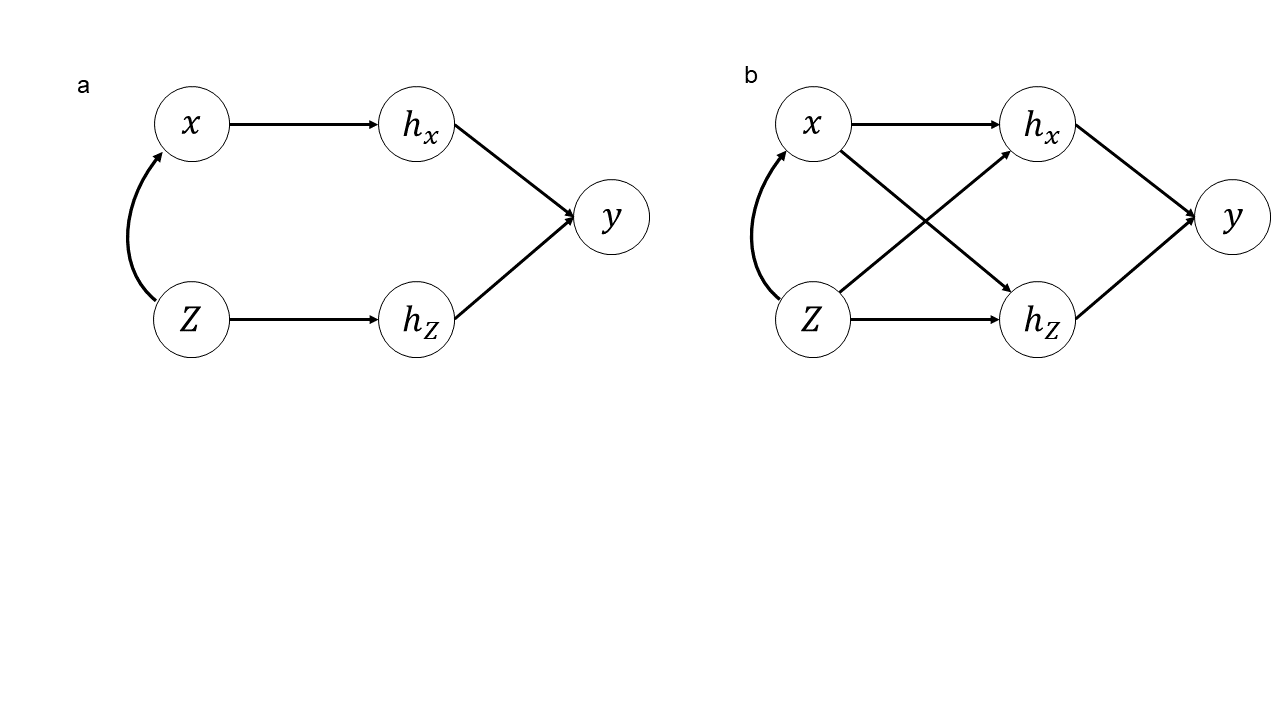


Figure S7. Causal structure diagram of two different generation processes, a stand for causal structure model in diffusion model, b stands for generative models of non-Markov processes such as autoencoders.

Table S1. The AUC results of Hela dataset

|  | Hela1 | Hela2 | Hela3 | Hela4 | Hela5 | Hela6 |
| --- | --- | --- | --- | --- | --- | --- |
| CDD | 0.701389 | 0.763889 | 0.736111 | 0.715278 | 0.756944 | 0.75 |
| GENIE3 | 0.573192 | 0.608466 | 0.518519 | 0.453263 | 0.416226 | 0.49382716 |
| ODEs | 0.385362 | 0.698413 | 0.417989 | 0.499118 | 0.354497 | 0.479717813 |
| PLSNet | 0.631393 | 0.663139 | 0.594356 | 0.698413 | 0.492063 | 0.686067019 |

The resulting AUCs for the gene regulatory network inference task in Hela dataset

Table S2. The AUC results of Dream4_knockdown size10 dataset

|  | Knock1 | Knock2 | Knock3 | Knock4 | Knock5 |
| --- | --- | --- | --- | --- | --- |
| CDD | 0.709804 | 0.678431 | 0.668627 | 0.708223 | 0.729167 |
| GENIE3 | 0.671111 | 0.533784 | 0.614222 | 0.719281 | 0.741452 |
| ODEs | 0.559111 | 0.516892 | 0.569778 | 0.611389 | 0.590812 |
| PLSNet | 0.624 | 0.587838 | 0.466667 | 0.48951 | 0.659188 |

The resulting AUCs for the gene regulatory network inference task in Dream4 dataset

Table S3. The AUC results of Dream4_timeseries size100 dataset

|  | Timeseries 1 | Timeseries 2 | Timeseries 3 | Timeseries 4 | Timeseries 5 |
| --- | --- | --- | --- | --- | --- |
| CDD | 0.678431 | 0.748512 | 0.537255 | 0.704244 | 0.833333 |
| GENIE3 | 0.824 | 0.633446 | 0.688 | 0.691309 | 0.80235 |
| ODEs | 0.627154 | 0.614551 | 0.658969 | 0.624919 | 0.689058 |
| PLSNet | 0.624 | 0.570946 | 0.562667 | 0.625375 | 0.590812 |

The resulting AUCs for the gene regulatory network inference task in Dream4 dataset

Table S4. The AUC results of disease dataset from TCGA[1]

|  | BLCA | BRCA | LIHC | PAAD |
| --- | --- | --- | --- | --- |
| GENIE3 | 0.481928 | 0.542998 | 0.524394 | 0.588595 |
| CDD | 0.662058 | 0.536262 | 0.511938 | 0.565228 |

The resulting AUCs for the gene regulatory network inference task in disease dataset

Table S5. Details of MRDataChallenge2019 dataset analysis using CDD.

| **CDD** | age-related macular degeneration | Alzheimer's disease | type 2 diabetes mellitus | ischemic stroke | aortic stroke | cardiac stroke | small vessel stroke | reference |
| --- | --- | --- | --- | --- | --- | --- | --- | --- |
| Acetoacetate | - | + | - | - | - | - | - | [2] |
| Acetate | - | - | - | - | - | - | - | [3] |
| Alanine | - | - | - | - | - | + | + | No paper |
| Albumin | - | + | - | - | - | - | - | [4] |
| ApoA1 | - | - | - | - | - | - | - | [5] |
| Citrate | - | - | - | - | - | + | + | [6] |
| Creatinine | - | - | - | + | - | - | + | [7, 8] |
| Esterified cholesterol | - | - | + | - | + | + | - | [9, 10] |
| Fatty acid length | - | + | - | - | - | + | - | [11] |
| Free cholesterol | - | + | + | - | - | - | - | [12] |
| Glucose | + | + | - | + | - | + | - | [13-16] |
| Glutamine | + | - | - | + | + | - | - | [17, 18] |
| Glycerol | - | - | - | + | - | - | - | [19] |
| Glycine | - | - | + | - | - | - | - |  |

Red indicates the incorrect results, and the green indicates the correct results. Light yellow indicates that there is no reference result yet (i.e., it is not possible to determine whether it is correct or incorrect). No paper means that there is a part of the research literature about this risk factors, but none of them related with disease. The “+” indicates that factor has causal effect on disease in the corresponding column, and “-” represents it does not have causal effect on disease in the corresponding column.

Table S6. Details of MRDataChallenge2019 dataset analysis using Mendelian randomization.

| **MR** | age-related macular degeneration | Alzheimer's disease | type 2 diabetes mellitus | ischemic stroke | aortic stroke | cardiac stroke | small vessel stroke | reference |
| --- | --- | --- | --- | --- | --- | --- | --- | --- |
| Acetoacetate | - | - | - | - | - | - | - | [2] |
| Acetate | + | - | + | - | - | - | - | [3] |
| Alanine | - | - | - | - | - | - | - | No paper |
| Albumin | - | - | - | - | - | - | - | [4] |
| ApoA1 | + | - | + | - | - | - | - | [5] |
| Citrate | - | - | - | + | - | - | + | [6] |
| Creatinine | - | - | - | - | - | - | - | [7, 8] |
| Esterified cholesterol | - | + | - | - | - | - | - | [9, 10] |
| Fatty acid length | - | - | - | + | - | - | - | [11] |
| Free cholesterol | - | - | + | - | - | - | - | [12] |
| Glucose | - | - | - | - | - | - | - | [13-16] |
| Glutamine | - | - | - | - | - | - | - | [17, 18] |
| Glycerol | - | - | - | - | - | - | - | [19] |
| Glycine | - | - | + | - | + | - | - |  |

Red indicates the incorrect results, and the green indicates the correct results. Light yellow indicates that there is no reference result yet (i.e., it is not possible to determine whether it is correct or incorrect). No paper means that there is a part of the research literature about this risk factors, but none of them related with disease. The “+” indicates that factor has causal effect on disease in the corresponding column, and “-” represents it does not have causal effect on disease in the corresponding column.

Table S7. Details of lipid and coronary artery disease analysis.

| **Class** | **Acronym** | **CDD** | **NS** | **reference** |
| --- | --- | --- | --- | --- |
| Acylcarnitine | AC | - | + | [20] |
| Cholesteryl ester | CE | - | - |  |
| Ceramide | Cer(d) | - | - |  |
| free cholesterol | COH | + | - | [21] |
| Dehydrocholesteryl ester | DE | + | - | unclear |
| Diacylglycerol | DG | + | + |  |
| Dihexosylceramide | Hex2Cer | - | - |  |
| Dihydroceramide | dhCer | + | + |  |
| GM1ganglioside | GM1 | - | - |  |
| GM3ganglioside | GM3 | + | - | unclear |
| Lysophosphatidylcholine | LPC | + | + |  |
| Lysoalkylphosphatidylcholine | LPC(O) | - | - |  |
| Lysoalkenylphosphatidylcholine | LPC(P) | - | - |  |
| Lysophosphatidylethanolamine | LPE | + | + |  |
| Lysoalkenylphosphatidylethanolamine | LPE(P) | + | - | unclear |
| Lysophosphatidylinositol | LPI | + | + |  |
| Monohexosylceramide | HexCer | - | - |  |
| Oxidized sterol ester | OxSpecies | - | - |  |
| Phosphatidylcholine | PC | - | - |  |
| Alkylphosphatidylcholine | PC(O) | - | - |  |
| Alkenylphosphatidylcholine | PC(P) | - | - |  |
| Phosphatidylethanolamine | PE | + | + |  |
| Alkylphosphatidylethanolamine | PE(O) | - | - |  |
| Alkenylphosphatidylethanolamine | PE(P) | + | - | Unclear |
| Phosphatidylglycerol | PG | - | + | No paper |
| Phosphatidylinositol | PI | + | - | [22] |
| Phosphatidylserine | PS | + | - | [23] |
| Sphingomyelin | SM | - | - |  |
| Sulfatide | SHexCer | - | - |  |
| Triacylglycerol | TG[NL] | + | + |  |
| Alkyldiacylglycerol | TG(O)[NL] | + | + |  |
| Trihexosylcermide | Hex3Cer | + | - | [24] |
| Ubiquinone | CoQ10 | + | + |  |

The data was presented whole lipidome data comprising 33 lipid major classes and 596 lipid minor classes. The population under investigation in the article was a European population, and the data was in the form of SNP data.

Unclear means that there is a lack of relevant literature on this lipid, and no paper means that there is a part of the research literature about this lipid, but none of them related with CAD. The “+” indicates that lipid major class has causal effect on coronary artery disease, and “-” represents it does not have causal effect on coronary artery disease.

Table S8. Potential factors and prostate cancer analysis.

| **Potential factors** | **AFR** | **CSA** | **EUR** |
| --- | --- | --- | --- |
| Albumin | + | + | + |
| Alkaline phosphatase | - | - | + |
| Alanine aminotransferase | - | + | + |
| Apolipoprotein A | + | + | + |
| Apolipoprotein B | + | + | + |
| Aspartate aminotransferase | - | + | + |
| Direct bilirubin | - | + | + |
| Urea | - | + | + |
| Calcium | + | + | + |
| Cholesterol | + | + | + |
| Creatinine | + | + | - |
| C-reactive protein | - | + | + |
| Cystatin C | + | - | + |
| Gamma glutamyltransferase | - | + | + |
| Glucose | - | - | + |
| Glycated haemoglobin (HbA1c) | + | + | + |
| HDL cholesterol | + | - | + |
| IGF-1 | + | - | + |
| LDL direct | + | - | + |
| Lipoprotein A | - | + | + |
| Oestradiol | - | - | - |
| Phosphate | - | + | + |
| SHBG | - | + | + |
| Total bilirubin | + | + | + |
| Testosterone | - | - | + |
| Total protein | + | + | + |
| Triglycerides | + | - | + |

AFR means African ancestry, CSA means Central/South Asian ancestry, EUR means European ancestry. The “+” indicates that the hidden factor has causal effect on prostate cancer in the column population, and “-” represents it does not have causal effect on prostate cancer in the column population.

Table S9. Potential factors and colorectal cancer analysis.

| **Potential factors** | **AFR** | **CSA** | **EUR** |
| --- | --- | --- | --- |
| Albumin | - | - | - |
| Alkaline phosphatase | - | + | - |
| Alanine aminotransferase | + | + | + |
| Apolipoprotein A | + | - | + |
| Apolipoprotein B | + | - | - |
| Aspartate aminotransferase | + | + | - |
| Direct bilirubin | + | - | + |
| Urea | + | - | - |
| Calcium | - | - | - |
| Cholesterol | + | - | - |
| Creatinine | - | + | + |
| C-reactive protein | + | - | + |
| Cystatin C | - | - | - |
| Gamma glutamyltransferase | - | + | + |
| Glucose | - | + | + |
| Glycated haemoglobin (HbA1c) | - | + | - |
| HDL cholesterol | - | + | - |
| IGF-1 | - | + | - |
| LDL direct | + | - | + |
| Lipoprotein A | + | - | - |
| Oestradiol | + | - | - |
| Phosphate | - | - | + |
| SHBG | + | - | - |
| Total bilirubin | + | - | + |
| Testosterone | - | - | - |
| Total protein | - | - | + |
| Triglycerides | - | + | - |

AFR means African ancestry, CSA means Central/South Asian ancestry, EUR means European ancestry. The “+” indicates that the hidden factor has causal effect on colorectal cancer in the column population, and “-” represents it does not have causal effect on colorectal cancer in the column population.

Table S10. Potential factors and disorders of lipoid metabolism analysis.

| **Potential factors** | **AFR** | **CSA** | **EAS** | **MID** | **AMR** | **EUR** |
| --- | --- | --- | --- | --- | --- | --- |
| Albumin | - | + | + | + | + | + |
| Alkaline phosphatase | + | + | - | - | + | + |
| Alanine aminotransferase | + | + | + | - | - | + |
| Apolipoprotein A | + | - | + | - | - | + |
| Apolipoprotein B | + | - | - | + | + | + |
| Aspartate aminotransferase | + | + | - | - | - | + |
| Direct bilirubin | + | + | + | - | - | - |
| Urea | - | + | - | - | - | - |
| Calcium | + | + | + | + | - | - |
| Cholesterol | + | + | - | - | - | + |
| Creatinine | + | + | + | + | - | + |
| C-reactive protein | + | + | + | - | + | - |
| Cystatin C | - | + | + | + | + | - |
| Gamma glutamyltransferase | - | + | + | + | - | + |
| Glucose | + | - | - | + | + | + |
| Glycated haemoglobin (HbA1c) | + | + | + | + | + | + |
| HDL cholesterol | + | + | + | - | - | + |
| IGF-1 | + | + | - | + | - | + |
| LDL direct | + | + | - | - | + | + |
| Lipoprotein A | - | + | + | - | + | + |
| Oestradiol | + | + | - | + | + | + |
| Phosphate | + | - | + | - | - | + |
| SHBG | + | - | + | - | + | - |
| Total bilirubin | + | + | + | - | + | + |
| Testosterone | + | - | + | - | - | + |
| Total protein | - | + | + | + | + | + |
| Triglycerides | + | + | - | + | + | + |

AFR means African ancestry, CSA means Central/South Asian ancestry, EAS means East Asian ancestry, MID means Middle Eastern ancestry, AMR means Admixed American ancestry, EUR means European ancestry. The “+” indicates that the hidden factor has causal effect on disorders of lipoid metabolism in the column population, and “-” represents it does not have causal effect on disorders of lipoid metabolism in the column population.

Table S11. Potential factors and lipoma of the skin analysis.

| **Potential factors** | **AFR** | **CSA** | **EUR** |
| --- | --- | --- | --- |
| Albumin | + | + | - |
| Alkaline phosphatase | - | - | - |
| Alanine aminotransferase | - | + | + |
| Apolipoprotein A | - | + | - |
| Apolipoprotein B | + | - | - |
| Aspartate aminotransferase | + | + | + |
| Direct bilirubin | - | - | + |
| Urea | + | + | + |
| Calcium | - | - | + |

AFR means African ancestry, CSA means Central/South Asian ancestry, EUR means European ancestry. The “+” indicates that the hidden factor has causal effect on lipoma of the skin in the column population, and “-” represents it does not have causal effect on lipoma of the skin in the column population.

Table S12. Results and comparisons of gene regulation network inference.

| Dataset | CDD | AGRN | NSGRN | scLink |
| --- | --- | --- | --- | --- |
| Konck1 | **0.709804** | 0.702 | 0.6 | 0.508889 |
| Konck2 | **0.678431** | 0.507 | 0.548986486 | 0.483108 |
| Konck3 | 0.668627 | 0.613 | **0.736888889** | 0.466222 |
| Konck4 | **0.708223** | 0.625 | 0.614385614 | 0.326174 |
| Konck5 | 0.729167 | **0.735** | 0.693376068 | 0.474359 |
| Hela1 | **0.701389** | 0.583 | 0.486772 | 0.582892416 |
| Hela2 | **0.763889** | 0.62 | 0.35097 | 0.505291 |
| Hela3 | **0.736111** | 0.542 | 0.602293 | 0.589947 |
| Hela4 | **0.715278** | 0.534 | 0.701058 | 0.470018 |
| Hela5 | **0.756944** | 0.491 | 0.460317 | 0.473545 |
| Hela6 | **0.75** | 0.509 | 0.486772 | 0.593474 |

The resulting AUCs for the gene regulatory network inference task.

Table S13. Results of MRDataChallenge2019 dataset.

| Phenotypes | CDD | multiMR | MR |
| --- | --- | --- | --- |
| Acetoacetate | **+** | **-** | - |
| Acetate | - | - | - |
| Alanine | - | - | - |
| Albumin | + | + | - |
| ApoA1 | - | - | - |
| Citrate | - | - | - |
| Creatinine | - | - | - |
| Esterified cholesterol | - | - | + |
| Fatty acid length | **+** | **-** | - |
| Free cholesterol | - | - | - |
| Glucose | + | + | - |
| Glutamine | - | - | - |
| Glycerol | - | - | - |
| Glycine | - | - | - |

+ means there is a causal effect; - means there is none.

**Supplementary Material S1**

**Proposition 1**. For any $D \perp\xi$ where D$=\left( x,Z,y \right)$ and $\perp$ indicates the independence. $x$ and $y$ are one-dimensional causal and effect variables respectively, and $\xi$ represents Gaussian noise, $Z$ is the other observed variable set, then the set of variables $x$ satisfies the backdoor criterion with respect to ${(h}_{x}, y)$. $h_{x}$ represents the hidden layer from $x$. Then, with do-calculus on the hidden layer variable $h_{x}$ and our CDD algorithm, we can determine the causal relation from $x$ to $y$ in the sense of Judea Pearl causality [25].

**Proof of Proposition 1.** To demonstrate that the backdoor criterion holds with those assumptions, we need to demonstrate two key conditions. Firstly, $x$ has no descendant node of $h_{x}$. Secondly, $x$ blocks all paths between $h_{x}$ and $y$ leading to $h_{x}$. Actually, if $h_{x}$ is a child node of $x$, and $h_{x}$ has only $x$ as its descendant node, then the property described in the above proposition is indeed satisfied. Thus, with the assumption of hidden layers in our algorithm, we can prove that the backdoor criterion holds, and thus we can obtain the causal relation from $x$ to $y$ in this model.

Specifically, since the forward process of the diffusion model can be obtained by reparameterization techniques, the following formula can be obtained:

$$\begin{aligned} H=\sqrt{\bar{\alpha}_{t}}D+\sqrt{1-\bar{\alpha}_{t}}\xi\#\left( S1 \right) \end{aligned}$$

where $\xi$is Gaussian noise and $\bar{\alpha}_{t}$ is the combination coefficient. Through the equation, it can be found that the hidden layer $H$ is related to the initial data $D$ and a noise term. And since $D$ and $\xi$ are independent. Therefore, we can consider that the part of $x$ that we want to fix in $do(h_{x}$) is only related to $x$(Figure S1a) and can therefore be described as follows:

$$\begin{aligned} h_{x}=\sqrt{\bar{\alpha}_{t}}x+\sqrt{1-\bar{\alpha}_{t}}\xi\#\left( S2 \right) \end{aligned}$$

$$and x \perp\xi$$

When considering that $Z$ has causal effect on both $x$ and $y$, the causal diagram can be described as Figure S1b. When considering that Z has a causal effect on only one of $x$ and $y$ or neither of them, the causal diagram can be described as Figure S1a (In the case where we're primarily concerned with the relationship between $x$ and $y$). Define$D_{1}=(Z,x)$. Then the relation between $x$, $y$ and $Z$ can be transformed into the form of Figure S1c.

Hence, $h_{x}$ is a child node of $x$, and only associates with $x$. The causal diagram can be described as Figure S1. Thus $x(D_{1})$ blocks all paths between $h_{x}$ and $y$ leading to $h_{x}$, i.e., the backdoor criterion holds.

After the backdoor criterion is satisfied, the next step is to solve the do-calculus formula, and the solution procedure comes from OrphicX [26], i.e.,

$$\begin{aligned} P\left( y | do\left( h_{x1} \right) \right)=\sum_{x} P\left( y | h_{x},x \right)P\left( x \right) \#\left( S3 \right) \end{aligned}$$

$$=\sum_{D_{1}} P\left( y | h_{x},D_{1} \right)P\left( D_{1} \right)$$

$$\approx\frac{1}{N_{D_{1}}}\sum_{k=1}^{N_{D_{1}}} P(Y|h_{x},{D_{1}}^{\left( k \right)})$$

where k indexes the $N_{D_{1}}$ sampled node attribute matrices ${D_{1}}^{\left( k \right)}$ from the test dataset. This means we can sample${D_{1}}^{\left( k \right)}$ to obtain $y$. Through two do operations, i.e., once under the hypothesis of H0 and once under the hypothesis of H1, different ones are obtained. By comparing the relationship between y, the causality between x and y can be judged, which proved the proposition. The causality of H0 and H1 is further demonstrated in the following part. $\square$

**Causality of the H0 and H1 tests**

This section examines whether or not the assumptions of H0 and H1 can deduce the rationality of causality.

The additive noise model (ANM):

$$\begin{aligned} Y=f\left( X \right)+\xi\#\left( S4 \right) \end{aligned}$$

where $X$ is the dependent variable and $Y$ is the effect variable.$\xi$ is noise. $f$ represents function relation. ANM model provides the identification of the causal direction. On this basis, we propose the H0 and H1 assumptions. The prerequisite conditions that need to be met for the H0 and H1 hypotheses are as follows.

**Prerequisite conditions**: we consider all noises to be random and unpredictable. It means noise $\xi$ is independent of dependent variable $X$.

In the case of multiple variables, we propose the following four scenarios.

Case one: $X$ has no influence on $Y$, while $Z$ is another variable that has influence on $Y$(case one in Figure S2).

Case two: $X$ has an impact on $Y$, while$Z$ is not affected by other variables (Figure S2 case two).

Case three: $X$ has an impact on $Y$, and $Z$ is other variables affecting $Y$ (Figure S2 case three).

Case four: $X$ has no influence on$Y$, and $Z$ is other variables have no influence on $Y$ (Figure S2 case four).

For the case one, we want to show that $y_{pre1}$ is not closer from $Y$than$y_{pre2}$ is from $Y$. $y_{pre1}$ comes from the y predicted under the H1 assumption. And $y_{pre2}$ comes from the $Y$ predicted under the H0 assumption. In this condition

$$y_{pre2}=f_{2}\left( Z \right)$$

$$y_{pre1}=f_{1}\left( X,Z \right)$$

From ANM, we can get the relationship between variables expressed as

$$\begin{aligned} Y=f_{Y}\left( Z \right)+\xi_{y} \#\left( S5 \right) \end{aligned}$$

$$X=f_{X}\left( Z \right)+\xi_{X}$$

If $f_{X}$ is invertible, then

$$Z=f_{X}^{-1}\left( X-\xi_{X} \right)$$

$$\begin{aligned} Y=f_{Y}\left( f_{X}^{-1}\left( X-\xi_{X} \right) \right)+\xi_{y} \#\left( S6 \right) \end{aligned}$$

Since $\xi_{X}$ is completely unknown, it is impossible to completely restore $Z$ from $X$, so the above formula can be described as

$$Y=f_{Y}\left( f_{X}^{-1}\left( X \right) \right)+g\left( \xi_{X}^{'} \right)+\xi_{y}$$

So, if we want to know the relationship between $X$ and $Y$, then by formula (S4) we have for $y_{pre2}$: the optimal condition $f_{2}\left( Z \right)$= $f_{Y}\left( Z \right)$ and Y distance difference factor $\xi_{y}$. For $y_{pre1}$: since $X$is independent of $\xi_{y}$, there is no fitting of the information to $\xi_{y}$ by $X$. The distance using $X$ to fit $Y$ is $g\left( \xi_{X}^{'} \right)+\xi_{y}$ by formula(S6). That is, the distance difference between$f_{1}(Z,X)$*=* $f_{Y}\left( Z \right)$ and $Y$ in the optimal case is$\xi_{y}$.

Therefore $y_{pre1}$ is not closer from $Y$ than $y_{pre2}$ is from $Y$.

For the case two, we want to show that $y_{pre1}$ is closer to $Y$ than $y_{pre2}$. We can get the relationship between variables expressed as

$$\begin{aligned} Y=f_{Y}\left( X \right)+\xi_{y} \#\left( S7 \right) \end{aligned}$$

$$Z=f_{Z}\left( X \right)+\xi_{Z}$$

If $f_{Z}$ is invertible, then

$$\begin{aligned} X=f_{Z}^{-1}\left( Z-\xi_{Z} \right) \#\left( S8 \right) \end{aligned}$$

$$\begin{aligned} Y=f_{Y}\left( f_{Z}^{-1}\left( Z-\xi_{Z} \right) \right)+\xi_{y}\#\left( S9 \right) \end{aligned}$$

Since $\xi_{z}$ is completely unknown, it is impossible to completely restore $Z$ from $X$, so the above formula can be described as

$$\begin{aligned} Y=f_{Y}\left( f_{Z}^{-1}Z \right)+g\left( \xi_{Z} \right)+\xi_{y} \#\left( S10 \right) \end{aligned}$$

So, if we want to know the relationship between X and Y, then based on our algorithm we have

$y_{pre1}=$ $f_{H1}\left( Z,X \right) ; y_{pre2}=$ $f_{H0}\left( Z \right)$

where $f_{H0}$ and $f_{H1}$ represent the functions from H0 and H1 assumption in formula (1) and (2) respectively.

For $y_{pre2}$, the optimal case is formula (S10) since $\xi_{y}$ and $\xi_{Z}$ are independent of $Z$; For $y_{pre1}$, the optimal case is formula (S7), which is proved to be consistent with the case one.

Hence, the difference between $y_{pre2}$ and y is $g(\xi_{Z})+\xi_{y}$, and the difference between $y_{pre1}$ and $Y$ is $\xi_{y}$. Thus $y_{pre1}$ is closer to $Y$ than $y_{pre2}$.

If $f_{Z}$ is irreversible, then $X$ cannot be expressed, and $\xi_{z}$ cannot be expressed for $Y$. In the absence of other factors, we can use $X=g_{z} \left( Z \right)+\xi_{X}^{'}$ to replace formula (S8). And then get the same result as (S9).

For the case three, the proof is similar to second case, except that $X$ cannot be directly represented by $Z$ in the same way as irreversible.

For the case four, $Y$ is the island point, and the proof process is same as case one. The difference is that $Y$ doesn't fit well with or without $X$.

If an unobserved confounding factor exists, we can judge the case of A (Figure S3a) and B (Figure S3b) in some cases, and the case of C (Figure S3c) is not well judged by the theory at present. However, within the context of the problem under discussion, the likelihood of case C is often lower compared to case A and case B. For instance, in the gene regulatory network, there exists a limited number of unobserved genes that exclusively influence only two genes ($X$ and $Y$) without impacting the gene set $Z$.

For case A (Figure S3a), we can get the relationship between the variables.

$$Y=f_{Y}\left( Z \right)+g_{Y}\left( U \right)+\xi_{Y}$$

$$X=f_{X}\left( Z \right)+g_{X}\left( U \right)+\xi_{X}$$

$$Z=f_{Z}\left( U \right)+\xi_{Z}$$

So, if $f_{Z}$ is invertible, then

$$U=f_{Z}^{-1}\left( Z-\xi_{Z} \right)$$

$U$ can also represent as

$$U=g_{X}^{-1}\left( X-\xi_{X}-f_{X}\left( Z \right) \right)$$

Then we get the representation of $Y$ as follows

$$\begin{aligned} Y={f_{Y}\left( Z \right)+f}_{Y}\left( f_{Z}^{-1}\left( Z-\xi_{Z} \right) \right)+\xi_{Y}={f_{Y}\left( Z \right)+f}_{Y}\left( f_{Z}^{-1}\left( Z \right) \right)+ϴ\left( \xi_{Z} \right)+\xi_{Y} \#\left( S10 \right) \end{aligned}$$

$$\begin{aligned} Y={f_{Y}\left( Z \right)+f}_{Y}\left( g_{X}^{-1}\left( X-\xi_{X}-f_{X}\left( Z \right) \right) \right)+\xi_{Y} \\ ={f_{Y}\left( Z \right)+f}_{Y}\left( g_{X}^{-1}\left( X-f_{X}\left( Z \right) \right) \right)+ϴ\left( \xi_{X} \right)+\xi_{Y}\#\left( S11 \right) \end{aligned}$$

which is the same as case two (Figure S2 case two). Hence, the difference between $y_{pre2}$ and $Y$ is $ϴ(\xi_{Z})+\xi_{Y}$ , and the difference between $y_{pre1}$ and $Y$ is $ϴ(\xi_{X})+\xi_{Y}$. Although we cannot judge the distance between the two, if $y_{pre2}$ is closer than $y_{pre1}$, it must be shown that $X$has no causal effect on $Y$. Because it means that the first distance is smaller than the second means that $X$ must have no effect on $Y$, as shown in case one.

The proof procedure in case B is the same as in case A (Figure S3).

**The combination of do-calculus and diffusion models:**

Methodologically, the primary advantage of combining diffusion models with the do-calculus lies in the following: The forward process of diffusion models adheres to the properties of a Markov process [27, 28]. This ensures the relationship between the hidden layer and the input. Consequently, by manipulating the hidden layer, the conditions for applying the backdoor criterion are satisfied, and the do-calculus can be used to infer the causal relationships between variables. We assumed variables D$=\left( x,Z,y \right)\in R^{n}$ as the data domain, and their corresponding $H=(h_{x}, h_{Z}, h_{y})\in R^{n}$ are hidden layer variables. When we focus on the relationship from variable *x* to *y*, and the$t$ from 1 to T represents the diffusion step in the diffusion model. The forward process of the diffusion model can be expressed as follows:

$$\begin{aligned} P\left( h_{x} | x \right)=\prod_{t=1}^{T} p_{\theta_{1}}\left( x_{t} | x_{t-1} \right) . \#\left( S12 \right) \end{aligned}$$

where $\theta_{1}$ is the forward process parameter artificially given, $t$represents the state of diffusion, $h_{x}$ represents the hidden layer of $x$, and $x$ denotes a variable in the input data. Specifically, $x_{t}$​ only depends on the previous time step $x_{t-1}$​. When $t=T$, $x_{t}$​ corresponds to $h_{x}$​. Therefore, $h_{x}$​ is only dependent on $x$, and is conditionally independent of $Z$ given $x$ (as illustrated in Figure S7a). However, if a non-Markovian generative model such as an autoencoder is chosen instead of a diffusion model, the generation function between $h_{x}$​​ and $x$ would be confounded, making it impossible to ensure the conditional independence of $h_{x}$​ and $Z$ given $x$ (as shown in Figure S7b). Hence, only under the use of a generative model like the diffusion model does the variable $x$ satisfy the backdoor criterion concerning ($h_{x}$,$y$), and thus the intervention can be represented as follows:

$$\begin{aligned} P\left( y | do\left( h_{x} \right) \right)=\sum_{x} P\left( y | h_{x},x \right)P\left( x \right) .\#\left( S13 \right) \end{aligned}$$

At this point, the intervention operation is transformed into a solvable form. $y$ denotes the effect variable. $h_{x}$ represents the hidden layer of $x$. The solution process is provided in the supplementary materials (details in section “Proposition 1” in Supplementary Material S1). By applying the intervention (details in section “Do-calculus on the hidden layer” in Supplementary Material S1), we can determine the causal relationship between $h_{x}$ and $y$. From the causal structure model, it can also be inferred that $h_{x}$​ is independent of $Z$ given $x$, and similarly, $h_{Z}$​ is independent of $x$ given $Z$. If the intervention reveals a causal effect between $h_{x}$​ and $y$, since $h_{x}$​ is only influenced by $x$ and not by $Z$, we can conclude that there is also a causal effect between $x$ and $y$, and this effect is not confounded by $Z$.

**Do-calculus on the hidden layer:**

Do-calculus, proposed by Judea Pearl [29] , is a reasoning tool designed to infer causal relationships from observed data. In real-world scenarios, the presence of confounding factors often leads to situations where highly correlated variables do not necessarily have a causal relationship. Traditional probabilistic inference relies on conditional probability. However, conditional probability alone cannot provide insights into causality, i.e. it only reveals correlation or association. To address the problem of intervention, Judea Pearl introduced the do-calculus. Additionally, he formulated three key rules to determine whether causal effects can be inferred from observed data.

Rule 1: Insertion/Deletion of Observations. If the variable $Z$ is independent of y (i.e., $Z$ is independent of $y$ given $x$), then the probability distribution of $y$ will not change with $Z$.

$$P\left( y | do\left( x \right),Z \right)=P\left( y | do\left( x \right) \right)$$

Rule 2: Action/Observation Exchange. If the variable $Z$ satisfies the backdoor criterion for ($x$, $y)$, then:

$$P\left( y | do\left( x \right),Z \right)=P\left( y | x,Z \right)$$

Rule 3: Rule of Reversal. If the intervention variable $x$ is independent of another variable $y$ given some other variables $Z$, then we can add or remove the intervention:

$$P\left( y | do\left( x \right),Z \right)=P\left( y | Z \right)$$

where $x$ represents the dependent variable, $y$ represents the effect variable, and $Z$ represents the other variables.

Backdoor Criterion: Given a directed acyclic graph and an ordered pair of variables ($h_{x}$, $y$), a set of variables $x$ satisfies the backdoor criterion with respect to ($h_{x}$, $y$) if

1. $x$ contains no descendants of $h_{x}$​;
2. $x$ blocks every path between $h_{x}$​ and $y$ that contains an arrow pointing into $h_{x}$​.

If these conditions are met, then $x$ satisfies the backdoor criterion for ($h_{x}$, $y$), and the following holds:

$$\begin{aligned} P\left( y | do\left( h_{x} \right) \right)=\sum_{x} P\left( y | {do(h}_{x}),x \right)P\left( x\left| {do(h}_{x} \right) \right)\#\left( S14 \right) \end{aligned}$$

$$=\sum_{x} P\left( y | h_{x},x \right)P\left( x\left| {do(h}_{x} \right) \right)$$

$$=\sum_{x} P\left( y | h_{x},x \right)P\left( x \right)$$

where $x$ represents the dependent variable, $y$ represents the effect variable, and $h_{x}$ represents the hidden layer of $x$.

By applying the backdoor criterion, we transform the do-calculus into a probabilistic form. Then we utilize the method from OrphicX [26] to obtain a solution. The detailed solution process is provided in the Supplementary Material S1 Proposition 1.

**Supplementary Material S2**

**Other domain data sets**

We choose the classic cigarette dataset on the kaggle dataset. On this dataset, we used the expression of variables in the dataset to infer relationships between variables. We compared the results using our method CDD and the instrumental variable method, respectively. CDD does not require instrumental variables, and can correctly judge that price and income will affect sales volume, while tax cannot directly affect sales volume. The instrumental variable approach does not. And CDD can judge that tax will affect price and price will not affect tax. Instrumental variables misjudge interaction effects (Figure S4).

**The causality for the potential factors to disorders of lipoid metabolism:**

There are 27 potential disease factors and 6 race populations (Central/South Asian, African, East Asia, Middle Easter, Admixed American, and European populations) to be stored in the UK Biobank database for the disorders of lipoid metabolism data. We used the CDD method to calculate the causality from the potential disease factors to disorders of lipoid metabolism and estimate the potential impact of the disease factors on disorders of lipoid metabolism for the 6 populations. we found all the disease factors can be related with disorders of lipoid metabolism (Figure S5 and Table S10), but it is interesting that different disease factors can be related to the disorders of lipoid metabolism in different race populations (Figure S5 and Table S10). The Albumin and total protein were found to have no causal effect on disorders of lipoid metabolism outcomes in African but have effect on both other populations (Figure S5 and Table S10). Albumin is clinically associated with chronic inflammation such as malaria [30], and total protein variables are also clinically associated with some inflammation [31]. So, we speculate that disorders of lipoid metabolism may be related to the living environment of African people.

**The causality for the potential factors to lipoma of the skin:**

We used 10 potential disease factors and 3 race populations stored in the UK Biobank database to investigate their association with lipoma of the skin. We used the CDD method to calculate the causality from the disease factors to lipoma of the skin and estimate the potential impact of the disease factors on lipoma of the skin for the 3 populations (Figure S6 and Table S11). We found only Alkaline phosphatase and Aspartate aminotransferase have the same causal effect on 3 populations (Figure S6 and Table S11), others have different causal effect among the 3 populations. For example, we found Calcium and Direct bilirubin have causal effect on lipoma of the skin outcomes in European, while they did show a causal effect in African and Central/South Asian populations (Figure S6 and Table S11). The European population receives less light than the other two groups because of the high latitude, which affects calcium absorption. Direct bilirubin is often associated with hepatobiliary diseases relating to drinking [32]. Therefore, we speculate that the environment factors such as light exposure and drinking may influence the development of lipoma of the skin in European population.

**Latest comparison baselines:**

Since the CDD method infers causal relationships from observed data without requiring additional prior information, it contrasts with many current approaches used in gene regulatory network inference tasks, such as CellOracle [33], and Glue [34], which depend on additional background information, *e.g.*, transcription factor. When this information is missing, these methods struggle to perform well. Similarly, if the additional information is provided to these methods in a comparison with CDD, the comparison would be unfair to CDD. Therefore, we chose to compare CDD with recent gene regulatory network inference methods that rely solely on gene expression data: AGRN [35], NSRGRN [36], and scLink [37].

We selected Dream4_size10 and HeLa datasets with ground truth for comparison. The datasets include 11 datasets (dream4_knockdowns1, dream4_knockdowns2, dream4_knockdowns3, dream4_knockdowns4, dream4_knockdowns5, Hela1, Hela2, Hela3, Hela4, Hela5, and Hela6). The most commonly used evaluation metric, AUC, was selected for performance comparison.

Using CDD, AGRN, NSRGRN, and scLink, we inferred causal relationships between variables on these 11 datasets (Table S12). The AUC values achieved by CDD were superior to those of the other three methods on 9 for the datasets (Table S12). Specifically, on the Hela1, Hela5, and Hela6 datasets, the AUC values using CDD were at least 10% higher than those of the other methods (Table S12).

In the context of causality inference for diseases and phenotypes, the absence of gold-standard datasets means that we rely more on CDD to explore novel findings. These are discoveries that methods such as Mendelian randomization [38] (detail in subsection “Identifying genes causally associated with kidney disease using SNPs” in the main text) or other statistical approaches [39] (detail in subsection “Lipidomic data related to coronary artery disease” in the main text) are generally difficult to reveal these causal discoveries.

For the MRDataChallenge2019 dataset, we previously applied standard Mendelian randomization. We expanded the analysis by incorporating a multivariable Mendelian randomization method (multiMR). The Alzheimer's disease was chosen, when the *p*-value threshold of 0.05 was selected for significance test.

Using CDD, we discovered that acetoacetate, fatty acid length, and free cholesterol are all causally related to Alzheimer's disease, and these findings that were validated in the literature[2, 11, 12]. However, these associations were not simply identified using multivariable Mendelian randomization (Table S13).

**Reference**

1. Zhang Y, Li Q, Chang X, Chen L, Liu X: **Causal network inference based on cross-validation predictability**. *BioRxiv* 2023.

2. Wu X-J, Shu Q-Q, Wang B, Dong L, Hao B: **Acetoacetate Improves Memory in Alzheimer’s Mice via Promoting Brain-Derived Neurotrophic Factor and Inhibiting Inflammation**. 2022, **37**:15333175221124949.

3. Regillo CD, D'Amico DJ, Mieler WF, Schneebaum C, Beasley CH, Sullins GT: **Clinical Safety Profile of Posterior Juxtascleral Depot Administration of Anecortave Acetate 15 mg Suspension as Primary Therapy or Adjunctive Therapy with Photodynamic Therapy for Treatment of Wet Age-Related Macular Degeneration**. *Survey of Ophthalmology* 2007, **52**(1):S70-S78.

4. Boada M, López OL, Olazarán J, Núñez L, Pfeffer M, Paricio M, Lorites J, Piñol‐Ripoll G, Gámez JE, Anaya F *et al*: **A randomized, controlled clinical trial of plasma exchange with albumin replacement for Alzheimer's disease: Primary results of the AMBAR Study**. *Alzheimer's & Dementia* 2020, **16**(10):1412-1425.

5. Wu X, Yu Z, Su W, Isquith DA, Neradilek MB, Lu N, Gu F, Li H, Zhao X-Q: **Low levels of ApoA1 improve risk prediction of type 2 diabetes mellitus**. *Journal of Clinical Lipidology* 2017, **11**(2):362-368.

6. Luo C, Bian X, Zhang Q, Xia Z, Liu B, Chen Q, Ke C, Wu J-L, Zhao Y: **Shengui Sansheng San Ameliorates Cerebral Energy Deficiency via Citrate Cycle After Ischemic Stroke**. *Frontiers in Pharmacology* 2019, **10**.

7. Akoudad S, Sedaghat S, Hofman A, Koudstaal PJ, van der Lugt A, Ikram MA, Vernooij MW: **Kidney Function and Cerebral Small Vessel Disease in the General Population**. *International Journal of Stroke* 2015, **10**(4):603-608.

8. Peng R, Liu K, Li W, Yuan Y, Niu R, Zhou L, Xiao Y, Gao H, Yang H, Zhang C *et al*: **Blood urea nitrogen, blood urea nitrogen to creatinine ratio and incident stroke: The Dongfeng-Tongji cohort**. *Atherosclerosis* 2021, **333**:1-8.

9. Oliveira CP, Maranhão RC, Bertato MP, Wajchenberg BL, Lerario AC: **Removal from the plasma of the free and esterified forms of cholesterol and transfer of lipids to HDL in type 2 diabetes mellitus patients**. *Lipids in Health and Disease* 2012, **11**(1).

10. Shibuya Y, Chang CCY, Chang T-Y: **ACAT1/SOAT1 as a therapeutic target for Alzheimer's disease**. *Future Medicinal Chemistry* 2015, **7**(18):2451-2467.

11. Silman I, Oikawa N, Matsubara T, Fukuda R, Yasumori H, Hatsuta H, Murayama S, Sato T, Suzuki A, Yanagisawa K: **Imbalance in Fatty-Acid-Chain Length of Gangliosides Triggers Alzheimer Amyloid Deposition in the Precuneus**. *Plos One* 2015, **10**(3).

12. Martín-Montes A, Recuero M, Sastre I, Vilella E, Rosich-Estragó M, Atienza M, Cantero JL, Frank-García A, Bullido MJ: **Cholesterol dysregulation in peripheral blood mononuclear cells of Alzheimer's disease**. *Journal of Neuroimmunology* 2022, **373**.

13. An Y, Varma VR, Varma S, Casanova R, Dammer E, Pletnikova O, Chia CW, Egan JM, Ferrucci L, Troncoso J *et al*: **Evidence for brain glucose dysregulation in Alzheimer's disease**. *Alzheimer's & Dementia* 2017, **14**(3):318-329.

14. Lee H, Han K-D, Shin J: **Association between glycemic status and age-related macular degeneration: A nationwide population-based cohort study**. *Diabetes & Metabolism* 2023, **49**(3).

15. Wang H, Zhou K, Li W, Du J, Xiao J: **Ctnnb1 transcriptional upregulation compensates for Mdm2/p53-mediated β-catenin degradation in neutrophils following cardioembolic stroke**. *Gene* 2021, **766**.

16. Yang Y, Huang X, Wang Y, Leng L, Xu J, Feng L, Jiang S, Wang J, Yang Y, Pan G *et al*: **The impact of triglyceride-glucose index on ischemic stroke: a systematic review and meta-analysis**. *Cardiovascular Diabetology* 2023, **22**(1).

17. Lewin AS, Kersten E, Dammeier S, Ajana S, Groenewoud JMM, Codrea M, Klose F, Lechanteur YT, Fauser S, Ueffing M *et al*: **Metabolomics in serum of patients with non-advanced age-related macular degeneration reveals aberrations in the glutamine pathway**. *Plos One* 2019, **14**(6).

18. Wang X, Zhang L, Sun W, Pei L-l, Tian M, Liang J, Liu X, Zhang R, Fang H, Wu J *et al*: **Changes of Metabolites in Acute Ischemic Stroke and Its Subtypes**. *Frontiers in Neuroscience* 2021, **14**.

19. Chen C, Duan F, Xie Y, Wan Q, Liu H, Gong J, Huang L, Song Z: **Nuciferine attenuates acute ischemic stroke in a rat model: a metabolomic approach for the mechanistic study**. *Molecular Omics* 2022, **18**(8):765-778.

20. Karagiannidis E, Moysidis DV, Papazoglou AS, Panteris E, Deda O, Stalikas N, Sofidis G, Kartas A, Bekiaridou A, Giannakoulas G *et al*: **Prognostic significance of metabolomic biomarkers in patients with diabetes mellitus and coronary artery disease**. *Cardiovascular Diabetology* 2022, **21**(1).

21. Smuts CM, Weich HFH, Weight MJ, Faber M, Kruger M, Lombard CJ, Spinnler Benadé AJ: **Free cholesterol concentrations in the high-density lipoprotein subfraction-3 as a risk indicator in patients with angiographically documented coronary artery disease**. *Coronary Artery Disease* 1994, **5**(4):331-338.

22. Jing R, Zhong QQ, Long TY, Pan W, Qian ZX: **Downregulated miRNA-26a-5p induces the apoptosis of endothelial cells in coronary heart disease by inhibiting PI3K/AKT pathway**. 2019.

23. Xia W, Yu H, Wang G: **Coronary Artery Disease with Elevated Levels of HDL Cholesterol Is Associated with Distinct Lipid Signatures**. *Metabolites* 2023, **13**(6).

24. You Q, Peng Q, Yu Z, Jin H, Zhang J, Sun W, Huang Y: **Plasma lipidomic analysis of sphingolipids in patients with large artery atherosclerosis cerebrovascular disease and cerebral small vessel disease**. *Bioscience Reports* 2020, **40**(9).

25. Pearl JJSS: **Causal inference in statistics: An overview**. 2009, **3**:96-146.

26. Lin W, Lan H, Wang H, Li B: **OrphicX: A Causality-Inspired Latent Variable Model for Interpreting Graph Neural Networks**. *IEEE/CVF Conference on Computer Vision Pattern Recognition* 2022:13719-13728.

27. Ho J, Jain A, Abbeel P: **Denoising diffusion probabilistic models**. In: *Proceedings of the 34th International Conference on Neural Information Processing Systems; Vancouver, BC, Canada*. Curran Associates Inc. 2020: Article 574.

28. Luo CJapa: **Understanding diffusion models: A unified perspective**. 2022.

29. Pearl J: **Causal inference in statistics: An overview**. *Statistics Surveys* 2009, **3** 96-146, 151.

30. Kuraeiad S, Kotepui KU, Mahittikorn A, Masangkay FR, Wilairatana P, Suwannatrai AT, Thinkhamrop K, Wangdi K, Kotepui M: **Albumin levels in malaria patients: a systematic review and meta-analysis of their association with disease severity**. *Sci Rep* 2024, **14**(1):10185.

31. Novokmet M, Lukic E, Vuckovic F, Ethuric Z, Keser T, Rajsl K, Remondini D, Castellani G, Gasparovic H, Gornik O *et al*: **Changes in IgG and total plasma protein glycomes in acute systemic inflammation**. *Sci Rep* 2014, **4**:4347.

32. O'Malley SS, Gueorguieva R, Wu R, Jatlow PI: **Acute alcohol consumption elevates serum bilirubin: an endogenous antioxidant**. *Drug Alcohol Depend* 2015, **149**:87-92.

33. Kamimoto K, Hoffmann CM, Morris SAJB: **CellOracle: Dissecting cell identity via network inference and in silico gene perturbation**. 2020:2020.2002. 2017.947416.

34. Cao Z-J, Gao GJNB: **Multi-omics single-cell data integration and regulatory inference with graph-linked embedding**. 2022, **40**(10):1458-1466.

35. Alawad DM, Katebi A, Kabir MWU, Hoque MTJBA: **AGRN: accurate gene regulatory network inference using ensemble machine learning methods**. 2023, **3**(1):vbad032.

36. Liu W, Yang Y, Lu X, Fu X, Sun R, Yang L, Peng LJBiB: **NSRGRN: a network structure refinement method for gene regulatory network inference**. 2023, **24**(3):bbad129.

37. Li WV, Li YJG, Proteomics, Bioinformatics: **scLink: inferring sparse gene co-expression networks from single-cell expression data**. 2021, **19**(3):475-492.

38. Ueda M, Fukui K, Kamatani N, Kamitsuji S, Matsuo A, Sasase T, Nishiu J, Matsushita M: **GLUT9 as a potential drug target for chronic kidney disease: Drug target validation by a Mendelian randomization study**. *J Hum Genet* 2023, **68**(10):699-704.

39. Cadby G, Giles C, Melton PE, Huynh K, Mellett NA, Duong T, Nguyen A, Cinel M, Smith A, Olshansky G *et al*: **Comprehensive genetic analysis of the human lipidome identifies loci associated with lipid homeostasis with links to coronary artery disease**. *Nat Commun* 2022, **13**(1):3124.
